# CDCSI: a machine learning-based interpretable cell death and cellular senescence index for prognosis improvement, immune landscape characterization, and therapeutic response prediction in head and neck squamous cell carcinoma

**DOI:** 10.3389/fimmu.2026.1848488

**Published:** 2026-05-22

**Authors:** Xiaoqin Luo, Xian He

**Affiliations:** Department of Otolaryngology Head and Neck Surgery, The Affiliated Traditional Chinese Medicine Hospital, Southwest Medical University, Luzhou, Sichuan, China

**Keywords:** cell death and cellular senescence, head and neck squamous cell carcinoma, machine learning, prognosis, treatment response

## Abstract

**Background:**

Head and neck squamous cell carcinoma (HNSCC) was characterized by pronounced heterogeneity and therapeutic resistance, which posed challenges for effective clinical management. Regulated cell death (RCD) and cellular senescence (CS) were recognized as fundamental hallmarks of cancer, exerting effects on tumorigenesis, progression, and treatment sensitivity. However, the utility of cell death and cellular senescence (CDCS) in predicting prognosis and therapeutic response had not been fully established.

**Methods:**

In this study, we integrated five types of multi-omics data with ten clustering algorithms to identify two distinct CDCS subtypes. Subsequently, subtype-associated marker genes and 270 machine learning algorithms were employed to construct a CDCS index (CDCSI). The CDCSI was then validated and compared across various datasets, including clinical features, external datasets, and previously published signatures. Finally, we evaluated the associations between CDCSI and various therapeutic strategies and validated its robustness at multiple levels.

**Results:**

First, we identified two distinct CDCS subtypes in HNSCC using multi-omics data and ten clustering algorithms. These subtypes exhibited different molecular characteristics, prognoses, and treatment responses, with CDCS2 showing broad resistance to radiotherapy, chemotherapy, targeted therapy, and immunotherapy. By analyzing marker genes associated with the CDCS2 subtype, we identified subtype-associated marker genes and integrated 270 machine learning algorithms to construct an individualized predictive model. The Ridge+SuperPC algorithm demonstrated the best performance, resulting in the development of CDCSI, which included six genes (SERPINE1, THBS1, SRPX, FADD, SERINC3, and CSNK2A2). We validated CDCSI across various methods, including clinical features, external datasets, and previously published models, confirming its superior predictive performance. Further analysis revealed that patients with low CDCSI were more likely to respond to radiotherapy, chemotherapy, EGFR-targeted therapy, and immunotherapy, whereas patients with high CDCSI exhibited stronger therapeutic resistance. The interpretability of CDCSI was enhanced through SHAP analysis, and its expression patterns were verified at single-cell and spatial transcriptomic levels, as well as through qPCR and WB for mRNA and protein expression.

**Conclusion:**

In conclusion, through the integration of bioinformatics analyses and machine learning algorithms, we developed CDCSI as a robust and reliable signature for predicting prognosis, characterizing molecular features, and evaluating potential responses to radiotherapy, chemotherapy, targeted therapy, and immunotherapy in HNSCC. These findings suggested that CDCSI may provide an exploratory framework for individualized risk stratification and hypothesis generation regarding therapeutic response in HNSCC, but prospective clinical validation is required before clinical application.

## Introduction

Head and neck squamous cell carcinoma (HNSCC), arising from the mucosal epithelium of the oral cavity, pharynx, and larynx, remains one of the most common and lethal malignancies worldwide (1–4). Despite substantial advances in surgery, radiotherapy, chemotherapy, targeted therapy, and immune checkpoint blockade, the prognosis of patients with recurrent, metastatic, or locally advanced HNSCC remained unsatisfactory (2, 5–7). A considerable proportion of patients still experienced recurrence, distant metastasis, therapeutic resistance, or limited durable benefit from systemic treatment, reflecting the marked molecular and cellular heterogeneity of this disease (8–11). These challenges highlighted the urgent need for robust biomarkers that could improve prognostic stratification and guide individualized precision treatment in HNSCC.

Among the biological processes that may underlie this heterogeneity, regulated cell death (RCD) has emerged as a critical determinant of tumor behavior and therapeutic responsiveness (12–14). RCD comprises multiple genetically controlled forms of cell death, including apoptosis, necroptosis, pyroptosis, ferroptosis, and others, each of which differs in molecular mechanisms, inflammatory consequences, and immunological effects (15–18). In cancer, these RCDs were not only involved in eliminating damaged or transformed cells but also participated in shaping antitumor immunity, inflammatory signaling, and treatment response (18–20). Increasing evidence suggested that dysregulation of RCD could contribute to tumor progression, immune escape, and therapeutic resistance, indicating that the biological significance of cell death extended far beyond its conventional role in tumor suppression (21–23).

Cellular senescence (CS) represented another fundamental stress-response program with profound relevance to cancer biology (24–26). It was generally characterized by a stable and essentially irreversible cell-cycle arrest induced by diverse endogenous and exogenous stresses, including telomere attrition, DNA damage, oxidative stress, oncogene activation, and anticancer treatment (27–30). In addition to proliferative arrest, senescent cells acquired a senescence-associated secretory phenotype (SASP), which consisted of pro-inflammatory cytokines, chemokines, growth factors, and proteases capable of extensively remodeling the tumor microenvironment (TME) (31–34). Although CS was initially regarded as a barrier against malignant transformation, persistent senescent cells and chronic SASP activity were increasingly recognized as drivers of inflammation, immune dysfunction, tumor progression, and treatment failure (35–39).

Importantly, cell death and cellular senescence (CDCS) did not act as isolated biological events but rather as tightly interconnected cell-fate programs (40–43). In response to cellular stress, the balance between death and senescence was thought to depend on the magnitude and duration of injury as well as the intrinsic molecular context of the cell (44, 45). These two processes shared overlapping upstream regulatory pathways and could reinforce one another through inflammatory and paracrine signaling (46–48). For instance, SASP factors secreted by senescent cells could modulate apoptotic, necroptotic, pyroptotic, or ferroptotic responses in neighboring cells, whereas damage-associated signals released during regulated cell death could in turn sustain senescence-associated phenotypes and reshape the surrounding tissue environment (31, 32, 49). Such reciprocal interactions suggested that the integrated CDCS axis might play a central role in determining malignant phenotypes, TME heterogeneity, and therapeutic responsiveness in HNSCC (50–52).

However, previous studies had largely examined individual RCD modes or CS in isolation, and a systematic understanding of their integrated roles in HNSCC remained limited (53, 54). For example, SLC25A1 was upregulated in HNSCC and promoted cisplatin resistance by inducing H3K27ac-mediated cellular senescence. Mechanistically, SLC25A1 interacted with HSPD1 to increase citrate transport and cytosolic acetyl-CoA levels, thereby activating RANBP1, CDC45, and PES1 and enhancing senescence, stemness, and chemoresistance (55). Similarly, USP10 was highly expressed in HNSCC and was associated with poor prognosis, as it stabilized POLR2A and upregulated SLC7A11 transcription to suppress ferroptosis. Targeting or depleting USP10 increased the sensitivity of HNSCC cells to ferroptosis inducers, suggesting that the USP10-POLR2A-SLC7A11 axis represented a potential therapeutic target (56). Together, these findings suggested that both CS and RCD-related pathways contributed to HNSCC progression and therapy resistance. Nevertheless, how the crosstalk between RCD and CS collectively shaped malignant phenotypes, remodeled the TME, and influenced prognosis and treatment response had not been fully elucidated. As a result, the potential value of integrating these processes into a unified molecular framework for patient stratification and therapeutic prediction remained largely unexplored in HNSCC.

In the study, we characterized the CDCS landscape of HNSCC through integrative multi-omics and machine learning analyses. We identified two distinct CDCS subtypes and found that the CDCS2 subtype was associated with unfavorable prognosis and resistance to multiple therapeutic modalities. Based on the marker genes of CDCS2, we further developed a CDCS Index (CDCSI) using 270 combinations of machine learning algorithms and validated its prognostic and therapeutic relevance across multiple independent cohorts. Our results showed that a high CDCSI was associated with poor survival and reduced sensitivity to radiotherapy, chemotherapy, targeted therapy, and immunotherapy, suggesting that CDCSI-based stratification may provide a clinically useful framework for prognostic prediction and precision treatment in HNSCC.

## Materials and methods

### Data acquisition and preprocessing

To characterize CDCS-related molecular heterogeneity in HNSCC, we collected multi-omics data from the TCGA-HNSCC cohort, including mRNA, lncRNA, miRNA, DNA methylation, somatic mutation, and corresponding clinical data (57). To further validate the generalizability of our findings in independent cohorts, we retrieved clinical information and mRNA expression profiles for four HNSCC datasets (GSE27020, GSE41613, GSE42743, and GSE65858) from the GEO database (58–61). In addition, due to the limited availability of publicly accessible HNSCC immunotherapy cohorts with matched transcriptomic profiles and clinical response data, four immunotherapy-related cohorts, including GSE78220, GSE91061, GSE135222, and IMvigor210, were obtained from GEO and the R package IMvigor210CoreBiologies to explore the broader immunological relevance of CDCS patterns (62–66). These cohorts mainly consisted of melanoma, non-small cell lung cancer, and urothelial carcinoma patients treated with immune checkpoint blockade. Microarray datasets were preprocessed using the limma package for background correction, log2 transformation, and quantile normalization (67). Boxplots were generated to assess post-normalization expression distributions (68). RNA-sequencing data were converted to FPKM values. To minimize non-biological variation across GEO cohorts, batch effects were corrected using the ComBat function, and the corrected datasets were merged into a META cohort (69). Principal component analysis (PCA) was used to evaluate the effectiveness and stability of data integration (70).

To investigate CDCS-related heterogeneity at higher resolution, single-cell RNA sequencing (scRNA-seq) and spatial transcriptomic (ST) datasets were additionally obtained from GSE103322 and GSE208253, respectively (71, 72). CDCS-related gene sets were curated from REACTOME, Gene Ontology (GO), FerrDb, and previous studies (**Supplementary Table 1**) (40, 73). CDCS-related miRNAs and lncRNAs were identified by co-expression analysis (correlation coefficient > 0.4 and p < 0.001) (74). Human cancer cell line expression data were further retrieved from the Broad Institute CCLE project and the GDSC database (75, 76).

### Multi-omics identification of CDCS subtypes

To identify CDCS-related molecular subtypes, feature selection across multiple omics layers was first performed using the getElites function in the MOVICS package. For continuous variables, including CDCS-related mRNA, miRNA, lncRNA, and DNA methylation data, the parameter method = “cox” was used to identify prognostic features associated with survival (p < 0.05). For binary somatic mutation data, the parameter method = “freq” was used to select the top 5% most frequently mutated genes. These selected features from five omics dimensions were then used for integrative clustering.

The optimal number of clusters was estimated using the getClustNum function in MOVICS, which integrates the Clustering Prediction Index (CPI), gap statistics, and Silhouette score (77). To improve clustering robustness, 10 integrative clustering algorithms were applied, including CIMLR, ConsensusClustering, SNF, iClusterBayes, PINSPlus, moCluster, NEMO, IntNMF, COCA, and LRA (78). Their outputs were subsequently integrated using the getConsensusMOIC function to derive consensus CDCS subtypes (79).

Subtype reproducibility was assessed in external cohorts using subtype-specific biomarkers, and concordance was further evaluated using the Nearest Template Prediction (NTP) and Partition Around Medoids (PAM) classifiers. To elucidate subtype-specific regulatory mechanisms, transcriptional regulatory networks were constructed using the RTN package, including 23 induced/repressed target-associated transcription factors and 71 candidate chromatin-remodeling regulators (80). Functional differences among subtypes were further characterized by GO and KEGG enrichment analyses (81, 82).

### Assessment of treatment response across CDCS subtypes

To explore the therapeutic relevance of CDCS subtypes, we evaluated their associations with radiotherapy, chemotherapy, targeted therapy, and tumor microenvironment (TME)-related features. Radiotherapy-related activity was estimated using ssGSEA based on two signatures, cell cycle and DNA replication, given their close relationship with proliferative status and radiosensitivity (68). Drug sensitivity was predicted using the oncoPredict package for conventional chemotherapeutic agents (5-Fluorouracil, Cisplatin, Docetaxel, Gemcitabine, and Paclitaxel) and EGFR-targeted agents (Afatinib, AZD3759 (Zorifertinib), Erlotinib, Gefitinib, Lapatinib, Osimertinib, and Sapitinib) (83). To further characterize subtype-specific immune and stromal states, a broad range of published TME-related signatures, including those associated with T cells, B cells, cancer-associated fibroblasts (CAFs), macrophages, immune checkpoint genes, immune function, immunotherapy, immune suppression, immune exclusion, immune exhaustion, and epithelial-mesenchymal transition (EMT), were quantified using the IOBR package and curated literature resources (84, 85).

### Construction of the cell death and cellular senescence index

To quantify CDCS patterns at the individual level, we established a CDCSI. The TCGA-HNSCC cohort, which contained relatively complete treatment and follow-up information, was used as the training set, whereas the remaining cohorts served as validation sets. Because the CDCS2 subtype was associated with unfavorable prognosis and resistance to multiple therapeutic modalities, we first identified subtype-associated marker genes. Univariate Cox regression analyses were conducted in both the TCGA-HNSCC and META cohorts, and genes with p < 0.01 and hazard ratio (HR) > 1 across cohorts were retained as candidate genes. These genes were subsequently used for model construction. To identify the most robust prognostic model, 10 machine learning algorithms were incorporated, including CoxBoost, stepwise Cox, Lasso, Ridge, elastic net (Enet), survival-SVM, generalized boosted regression models (GBMs), supervised principal components (SuperPC), plsRcox, and random survival forest (RSF) (68). Detailed information on the specific R packages, functions, and parameter settings used for machine learning implementation was provided in the ML.R file deposited in the GitHub repository (https://github.com/JYfantast/HNSCC). A total of 270 algorithmic combinations were evaluated. Model performance was assessed by the concordance index (C-index) in the training cohort and all validation cohorts, and the model with the highest average C-index was selected as the final CDCSI model.

### Validation and prognostic evaluation of CDCSI

The CDCSI was calculated for each sample in the training and validation cohorts, and patients were stratified into high-CDCSI and low-CDCSI groups. Prognostic differences between groups were assessed using Kaplan-Meier survival analysis. To benchmark CDCSI against existing prognostic models, we retrieved 88 published HNSCC prognostic signatures and calculated corresponding scores using their original coefficients (78). The prognostic performance of CDCSI and these signatures was compared across cohorts using the C-index. To determine whether CDCSI independently predicted prognosis, univariate and multivariate Cox regression analyses were performed incorporating conventional clinical variables. In addition, a nomogram integrating CDCSI with standard clinical characteristics was developed to facilitate individualized survival prediction.

### Association of CDCSI with therapeutic sensitivity

To further assess the clinical utility of CDCSI, we analyzed its correlation with radiotherapy-, chemotherapy-, and targeted therapy-related signatures. These analyses were performed to determine whether CDCSI could capture individual-level therapeutic vulnerability. The relationship between CDCSI and immunotherapy efficacy was further evaluated in multiple immunotherapy cohorts, including GSE78220, GSE135222, GSE91061, and IMvigor210. Associations with both prognosis and immunotherapy response were examined. In addition, the Tumor Immune Dysfunction and Exclusion (TIDE) framework was applied to estimate immune escape potential and the likelihood of response to immune checkpoint blockade (86). TIDE was used as a computational surrogate for estimating immune evasion and potential response to immune checkpoint blockade, rather than as direct clinical response evidence.

### Model interpretability analysis

To improve model transparency, Shapley Additive Explanations (SHAP) analysis was performed. SHAP is a game theory-based approach that quantifies the contribution of each feature to model output across all possible feature combinations. By calculating the Shapley value of each gene, we identified the features with the greatest impact on CDCSI prediction, thereby improving biological interpretability and strengthening the reliability of the model.

### Single-cell and spatial transcriptomic validation

To validate CDCSI at higher biological resolution, we integrated scRNA-seq and ST analyses. The GSE103322 scRNA-seq dataset, comprising 5,902 cells from 18 HNSCC patients, was processed using Seurat (version 5.2.1), including normalization, dimensionality reduction, clustering, and cell type annotation (71, 87). The ssGSEA algorithm was then used to calculate CDCSI across cell types, and the results were visualized using UMAP and violin plots. The GSE208253 ST dataset, comprising 12 fresh-frozen surgical resection samples from 10 HNSCC patients, was processed using Seurat (version 5.2.1), including quality control, normalization, dimensionality reduction, batch correction, and clustering (72). To infer the cellular composition of spatial spots, RCTD-based deconvolution was performed using GSE103322 as the reference scRNA-seq dataset (88). CDCSI were then computed in the spatial context using ssGSEA and visualized with spatial distribution and correlation plots.

### Cell lines and culture

The NP69 and FaDu cell lines were purchased from ATCC. The NP69 cell was cultured in DMEM medium (Gibco, Carlsbad, CA, USA) supplemented with 10% FBS (Gibco, Carlsbad, CA, USA) at 37 °C in a 5% CO_2_ incubator. The FaDu/DDP cell line was built by slowly increasing the concentration of cisplatin (Sigma, MO, USA) exposed to parental cells. The FaDu and FaDu/DPP cells were cultured in sodium pyruvate (1 mM), streptomycin (100 μg/ml), penicillin G (100 U/ml) and 10% FBS containing MEM medium in a humidified 37 °C, 5% CO_2_ incubator.

### RNA extraction and RT-qPCR

Total RNA was extracted by Trizol (Takara), and RNA quality and concentration were detected by the Nanodrop 2000 (Thermo) spectrophotometer, 1000 ng of total RNA was reversely transcribed into cDNA using the PrimeScript RT kit (Takara).The reaction conditions were set as follows: pre-denaturation at 95 °C for 30 s, denaturation at 95 °C for 5 s, annealing at 58 °C for 30 s and extension at 72 °C for 5 s, with a total of 40 cycles. The SYBR Premix Ex TaqTM reagent (Takara) was used for RT-qPCR analysis on Bio-Rad CFX96 (Bio-RAD) system. All RT-qPCR experiments were performed with three independent biological replicates, and each biological replicate was tested in triplicate technical replicates. The average Ct value of the technical replicates was used for data analysis. The relative quantitative data of mRNA was analyzed by the 2-ΔΔCt method. β-Actin was used as an internal reference. The primer sequences are shown in **Supplementary Table 2**.

### Western blot

The cells were lysed by RIPA with protease inhibitor (Selleck Chemicals, Texas, USA) at 4 °C for 30 mins and centrifuged at 14000 rpm for 30 mins. Then the supernatant was collected and boiled with protein loading buffer at 100 °C for 5 mins. The same amount (30 µg) of protein was separated by SDS-PAGE and electrotransferred onto a PVDF membrane by the rapid transfer film instrument Trans-Blot Turbo (Bio-Rad, CA, USA). The membrane was blocked with 5% milk for 2 h and then incubated with the primary antibody against SERPINE1 (13801-1-AP, Proteintech, Wuhan, China), SERINC3 (20267-1-AP, Proteintech, Wuhan, China), CSNK2A2 (10606-1-AP, Proteintech, Wuhan, China), FADD (14906-1-AP, Proteintech, Wuhan, China), THBS1 (18304-1-AP, Proteintech, Wuhan, China), and Tubulin (66031-1-Ig, Proteintech, Wuhan, China) overnight at 4 °C. After that, it was incubated with the secondary antibody at room temperature for 1.5 h. Finally, the membrane was inspected by ECL Western Blotting Substrate Kit (Millipore, USA). All Western blot analyses were performed with three independent biological replicates (three separately prepared cell lysates). Representative blots from one of the three replicates are shown.

### Statistical analysis

All wet-lab experiments were performed with at least three independent biological replicates (n ≥ 3). All analyses and figure generation were performed using R software (version 4.3.3). For two-group comparisons, the unpaired Student’s t test was used for normally distributed variables, whereas the Wilcoxon rank-sum test was applied for non-normally distributed variables. Survival differences were assessed using the Kaplan-Meier method and compared by the log-rank test. All statistical procedures were selected to ensure rigorous evaluation of group differences, survival outcomes, and model performance.

## Results

### Recognition of CDCS subtypes by multi-omics analysis and machine learning

**Figure 1:**
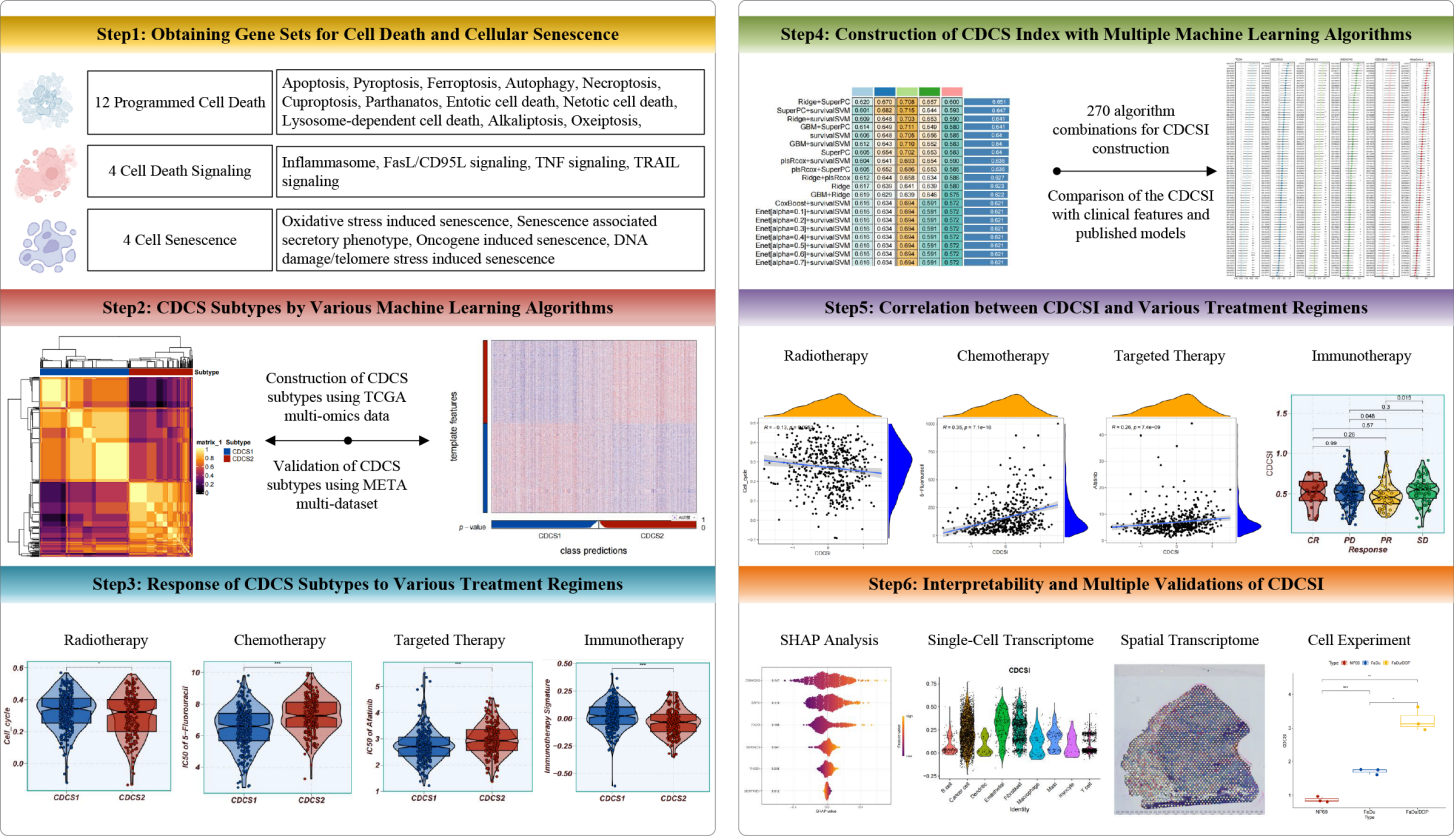
The overall workflow of this study.

**Figure 2:**
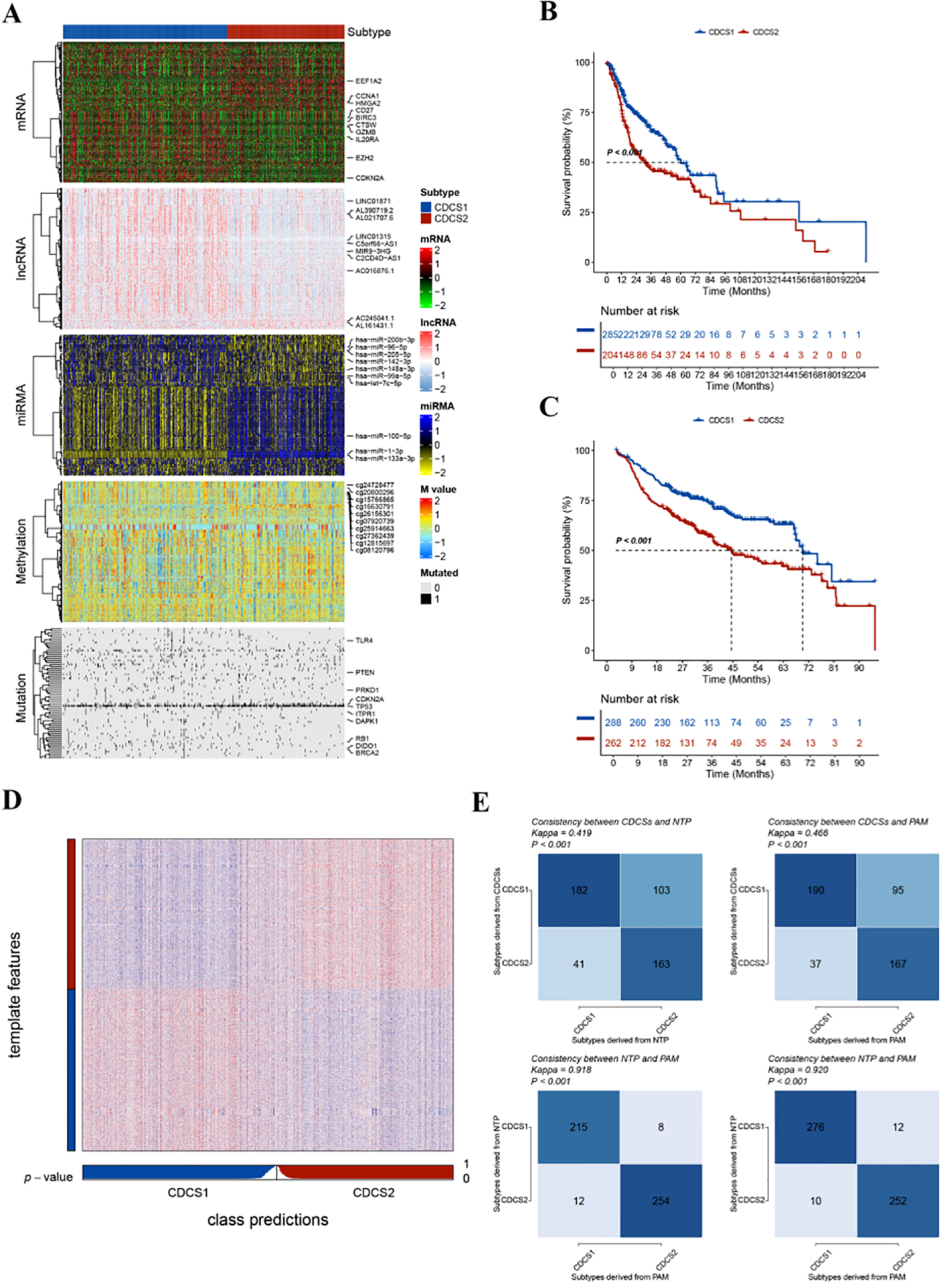
Identification and validation of CDCS subtypes by multi-omics analysis and machine learning. **(A)** CDCS multi-omics consensus clustering analysis visualization. **(B, C)** Survival analysis indicated that CDCS2 was associated with a poorer prognosis than CDCS1 in both the TCGA and META cohorts. **(D)** Validating CDCS subtypes in the META cohort using heatmaps. **(E)** The TCGA and META cohorts’ CDCS and NTP, CDCS and PAM, and NTP and PAM consistency.

The overall workflow of this study is illustrated in **Figure 1**. After data preprocessing and normalization, PCA was first performed to evaluate the influence of batch effects across cohorts. Compared with the distribution before correction, the corrected expression profiles showed improved sample comparability and reduced non-biological variation, supporting the reliability of subsequent integrative analyses (**Supplementary Figures 1A, B**). To identify robust CDCS-related molecular patterns in HNSCC, we applied multiple complementary approaches to determine the optimal cluster number, including the silhouette score, gap statistic, and clustering prediction index. These analyses consistently supported the presence of two major CDCS-related molecular subtypes (**Supplementary Figures 2A-C**). Based on these criteria, two robust CDCS-related subtypes were identified using ten integrative multi-omics clustering algorithms. Subsequently, a consensus ensemble approach was employed to integrate the clustering results, which revealed distinct and consistent molecular expression patterns across multiple omics layers, including CDCS-related mRNAs, DNA methylation profiles, somatic mutations, lncRNAs, and miRNAs (**Figure 2A**; **Supplementary Figure S2D**). Importantly, survival analysis demonstrated that the CDCS2 subtype was associated with a significantly poorer overall survival (OS) compared to the other subtype (p < 0.001), indicating its potential clinical relevance (**Figure 2B**).

### Validation of CDCS subtypes in external datasets

To examine whether the identified CDCS subtypes were reproducible beyond the training cohort, we further validated the classification in external datasets. A total of 2,000 subtype-specific upregulated genes were selected as classifier signatures for each subtype and applied to the META cohort. Consistent with the TCGA-HNSCC cohort, patients assigned to the CDCS2 subtype in the META cohort had significantly worse overall survival than those assigned to CDCS1 (p < 0.001; **Figure 2C**). The heatmap of subtype-specific genes in the META cohort showed that the expression patterns of classifier genes remained clearly distinguishable between the two subtypes, supporting the molecular stability of the classification (**Figure 2D**). In addition, both NTP and PAM methods were used to independently evaluate subtype assignment consistency. The strong concordance among consensus clustering, NTP, and PAM classification in both TCGA and META cohorts confirmed that the CDCS subtype system was robust and reproducible across independent datasets (p < 0.001; **Figure 2E**).

### Molecular characterization of CDCS subtypes

**Figure 3:**
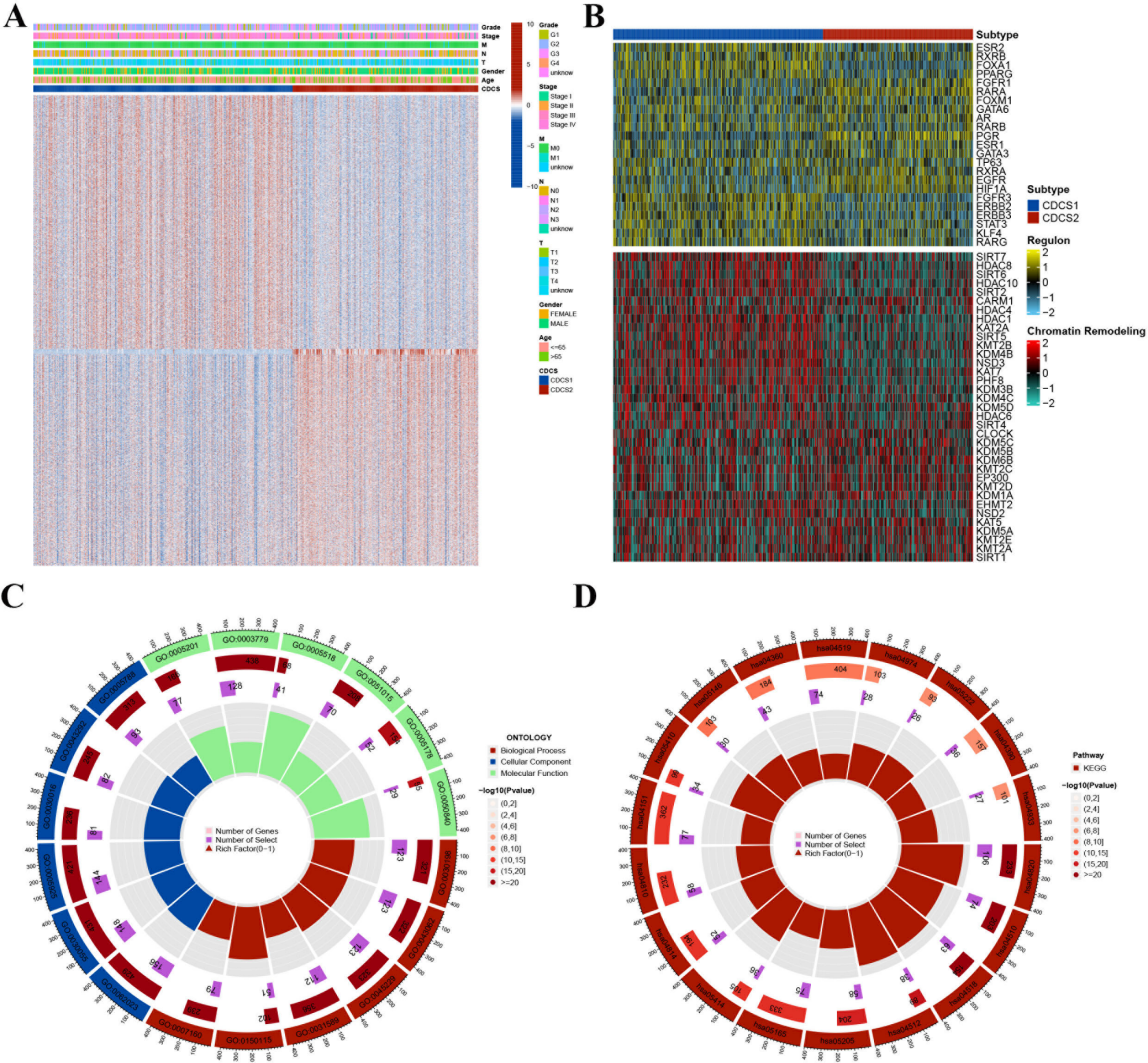
Molecular characterization of CDCS subtypes. **(A)** A heatmap was used to assess the association between CDCS subtypes and clinical characteristics. **(B)** TF networks and chromatin remodeling regulators were analyzed, revealing potential upstream regulatory mechanisms underlying subtype-specific gene expression. **(C)** The GO analysis for the CDCS2-related genes. **(D)** The KEGG analysis for the CDCS2-related genes.

To further clarify the biological differences between CDCS1 and CDCS2, we analyzed their associations with clinical characteristics and molecular regulatory programs. The clinical heatmap suggested that CDCS subtype classification was associated with distinct clinicopathological and molecular features (**Figure 3A**). To identify potential upstream regulatory mechanisms, transcription factor networks and chromatin remodeling regulators were further analyzed. These analyses revealed subtype-specific regulatory activities, suggesting that the two CDCS subtypes may be driven by different transcriptional and epigenetic regulatory programs (**Figure 3B**). GO enrichment analysis revealed that subtype-specific genes were closely associated with tumor microenvironment remodeling and invasive behavior. These genes were predominantly involved in processes such as ECM organization, cell-matrix and cell-substrate adhesion, junction assembly, wound healing, and ameboidal-type cell migration, reflecting their contribution to tissue remodeling and cell motility. At the subcellular level, they localized primarily to collagen-rich ECM, focal adhesions, cell-substrate junctions, actin filament bundles, and the leading edge of cells, with additional enrichment in contractile structures such as myofibrils, sarcomeres, and the endoplasmic reticulum lumen. Functionally, these genes mediated ECM structural activity and interactions with actin, collagen, integrins, cadherins, growth factors, and glycosaminoglycans, underscoring their roles in cytoskeletal regulation, matrix interaction, and cell adhesion (**Figure 3C**; **Supplementary Table 3**). KEGG pathway analysis further revealed that these genes were involved in focal adhesion, integrin signaling, ECM-receptor interaction, proteoglycans in cancer, regulation of the actin cytoskeleton, PI3K-Akt signaling, and human papillomavirus infection. Additional enrichment was observed in pathways related to cytoskeletal dynamics in muscle cells, motor proteins, and dilated cardiomyopathy (**Figure 3D**; **Supplementary Table 4**).

### Evaluation of treatment response across CDCS subtypes

**Figure 4:**
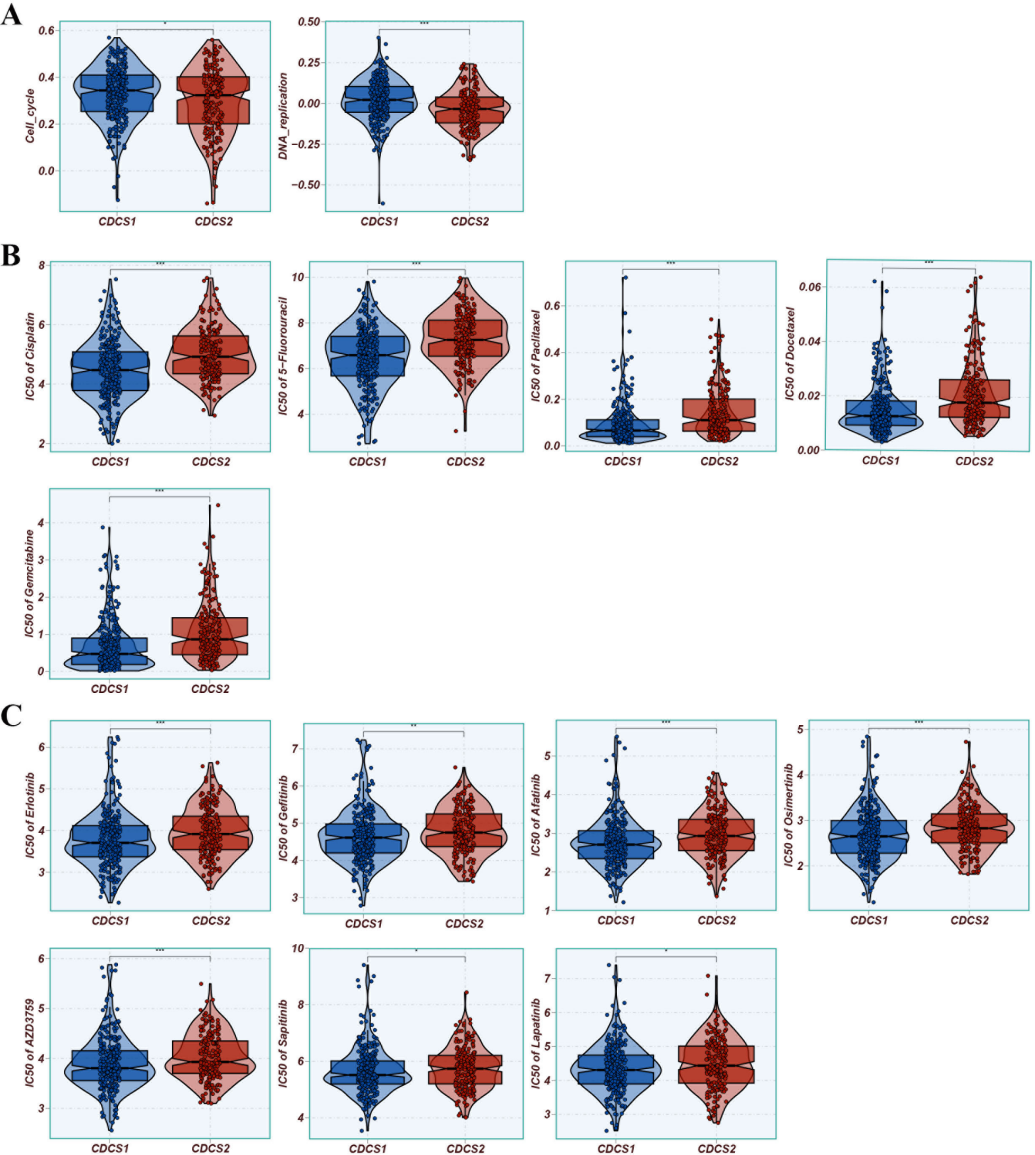
Evaluation of response to chemotherapy, radiation, and EGFR-targeted treatment across CDCS subtypes. **(A)** Analysis of radiotherapy-predictive biomarkers, specifically cell cycle and DNA replication signatures, revealed significant enrichment in the CDCS1 subtype, suggesting a greater suitability of these patients for radiotherapy. **(B)** CDCS1 patients exhibited enhanced predicted sensitivity to five conventional chemotherapeutic agents (5-Fluorouracil, Cisplatin, Docetaxel, Gemcitabine, and Paclitaxel), indicating a favorable response to chemotherapy. **(C)** CDCS1 patients demonstrated increased sensitivity to seven EGFR-targeted agents, including Afatinib, AZD3759 (Zorifertinib), Erlotinib, Gefitinib, Lapatinib, Osimertinib, and Sapitinib, supporting potential benefit from EGFR-targeted therapy. *, p < 0.05; **, p < 0.01; ***, p < 0.001.

**Figure 5:**
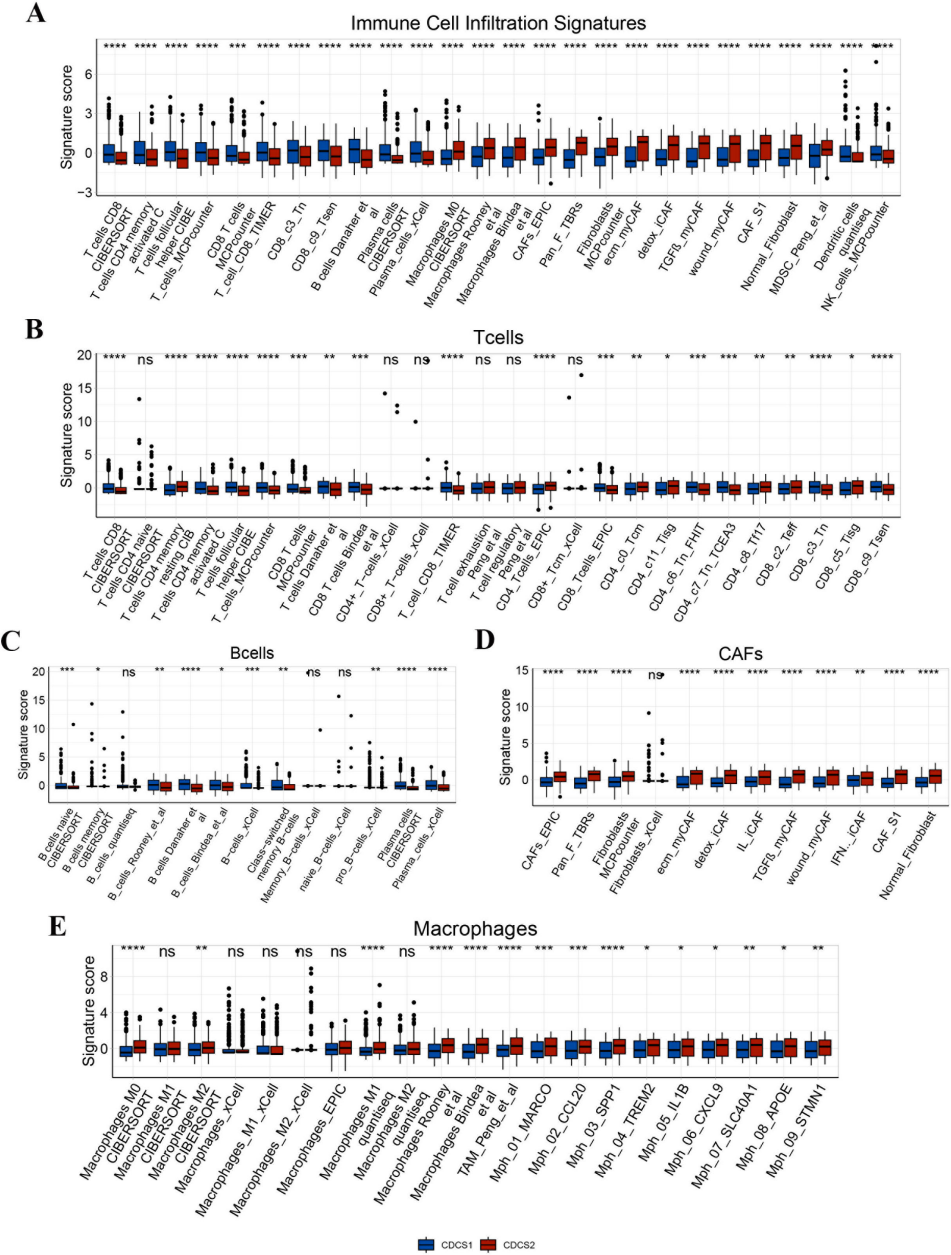
Assessment of the tumor microenvironment in different CDCS subtypes. **(A–E)** Comparison of tumor microenvironment cell infiltration between CDCS1 and CDCS2. CDCS1 showed higher enrichment of immune-active cell populations, including T cells and B cells, indicating a more inflamed immune microenvironment. In contrast, CDCS2 was characterized by higher enrichment of cancer-associated fibroblasts, suggesting stromal activation and an immunosuppressive tumor microenvironment. ns, p > 0.05; *, p < 0.05; **, p < 0.01; ***, p < 0.001; ****, p < 0.0001.

**Figure 6:**
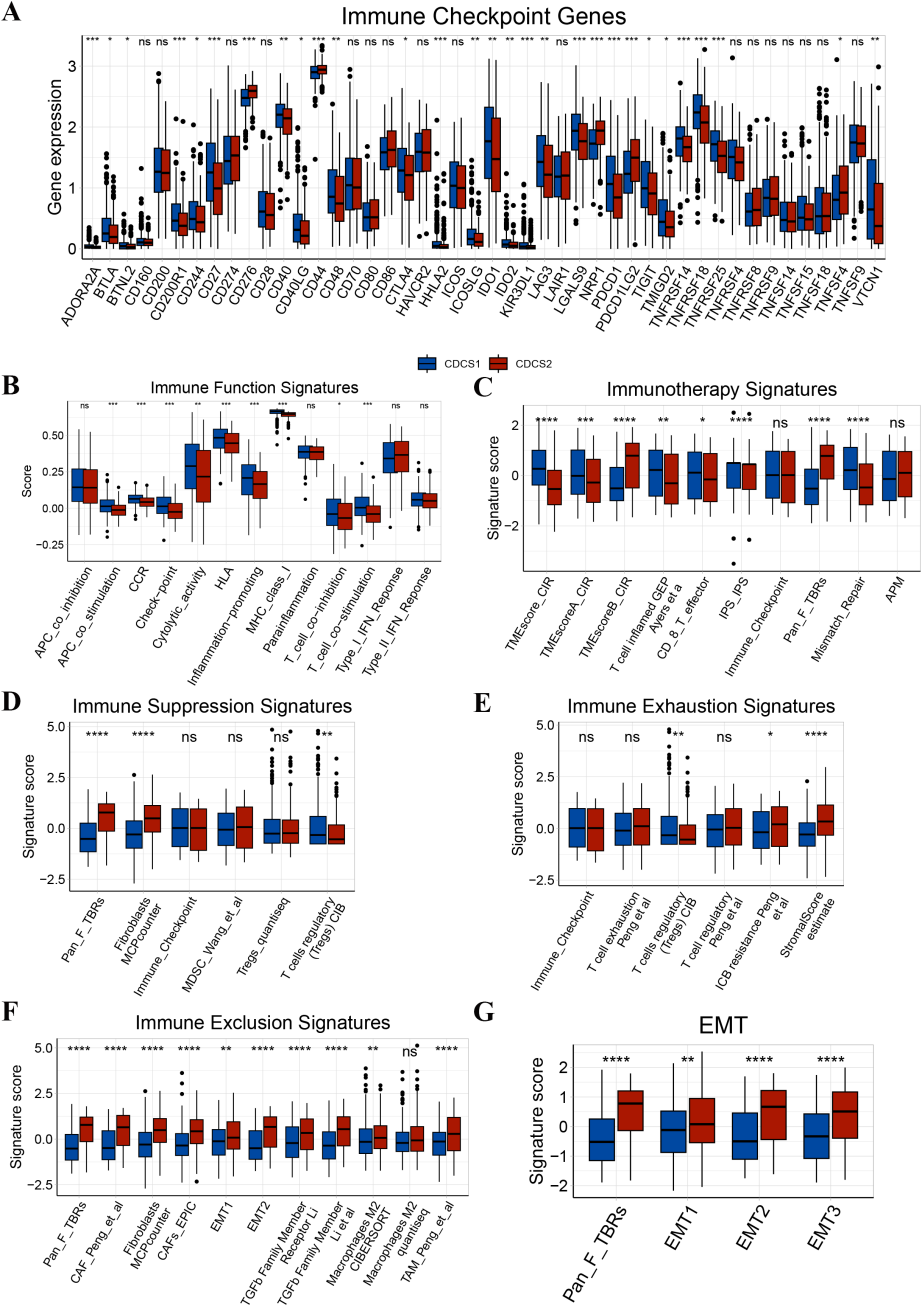
Evaluation of response to immunotherapy across CDCS subtypes. **(A-G)** Comparison of immune-related signatures between CDCS1 and CDCS2. CDCS2 exhibited higher enrichment of immune suppression, immune exclusion, immune exhaustion, and epithelial-mesenchymal transition signatures. In contrast, CDCS1 showed higher enrichment of immunotherapy-related signatures, immune function markers, and immune checkpoint genes. These results suggest that CDCS1 may represent an immune-active subtype more likely to benefit from immunotherapy, whereas CDCS2 may represent an immune-suppressed or immune-excluded subtype. ns, p > 0.05; *, p < 0.05; **, p < 0.01; ***, p < 0.001; ****, p < 0.0001.

We next investigated the potential therapeutic implications of CDCS subtypes across standard treatment modalities, including radiotherapy, chemotherapy, targeted therapy, and immunotherapy. Analysis of radiotherapy-predictive biomarkers, specifically cell cycle and DNA replication signatures, revealed significant enrichment in the CDCS1 subtype, suggesting a greater suitability of these patients for radiotherapy (**Figure 4A**). Consistently, CDCS1 patients also exhibited enhanced predicted sensitivity to five conventional chemotherapeutic agents (5-Fluorouracil, Cisplatin, Docetaxel, Gemcitabine, and Paclitaxel), indicating a favorable response to chemotherapy (**Figure 4B**). Similarly, this subtype demonstrated increased sensitivity to seven EGFR-targeted agents, including Afatinib, AZD3759 (Zorifertinib), Erlotinib, Gefitinib, Lapatinib, Osimertinib, and Sapitinib, supporting potential benefit from EGFR-targeted therapy (**Figure 4C**). Collectively, these findings suggest that patients with CDCS1 are more likely to respond to radiotherapy, chemotherapy, EGFR-targeted treatments, and immunotherapy. To further evaluate the immunotherapeutic relevance of CDCS subtypes, we performed comprehensive analyses using the IOBR package. CDCS1 exhibited significant enrichment of T cells and B cells, reflecting an active immune microenvironment, whereas CAFs were predominantly enriched in CDCS2, indicating an immunosuppressive context (**Figures 5A–E**). Additional analyses showed that CDCS2 was characterized by pronounced enrichment of immune suppression, immune exclusion, and EMT signatures, whereas CDCS1 displayed higher levels of immunotherapy-related signatures, immune function markers, and immune checkpoint genes (ICGs) (**Figures 6A–G**). These results collectively highlight the distinct therapeutic vulnerabilities of the two CDCS subtypes, with CDCS1 patients likely to benefit from multiple treatment modalities, while CDCS2 patients exhibit features of resistance and immune evasion.

### Construction of the cell death and cellular senescence index

**Figure 7:**
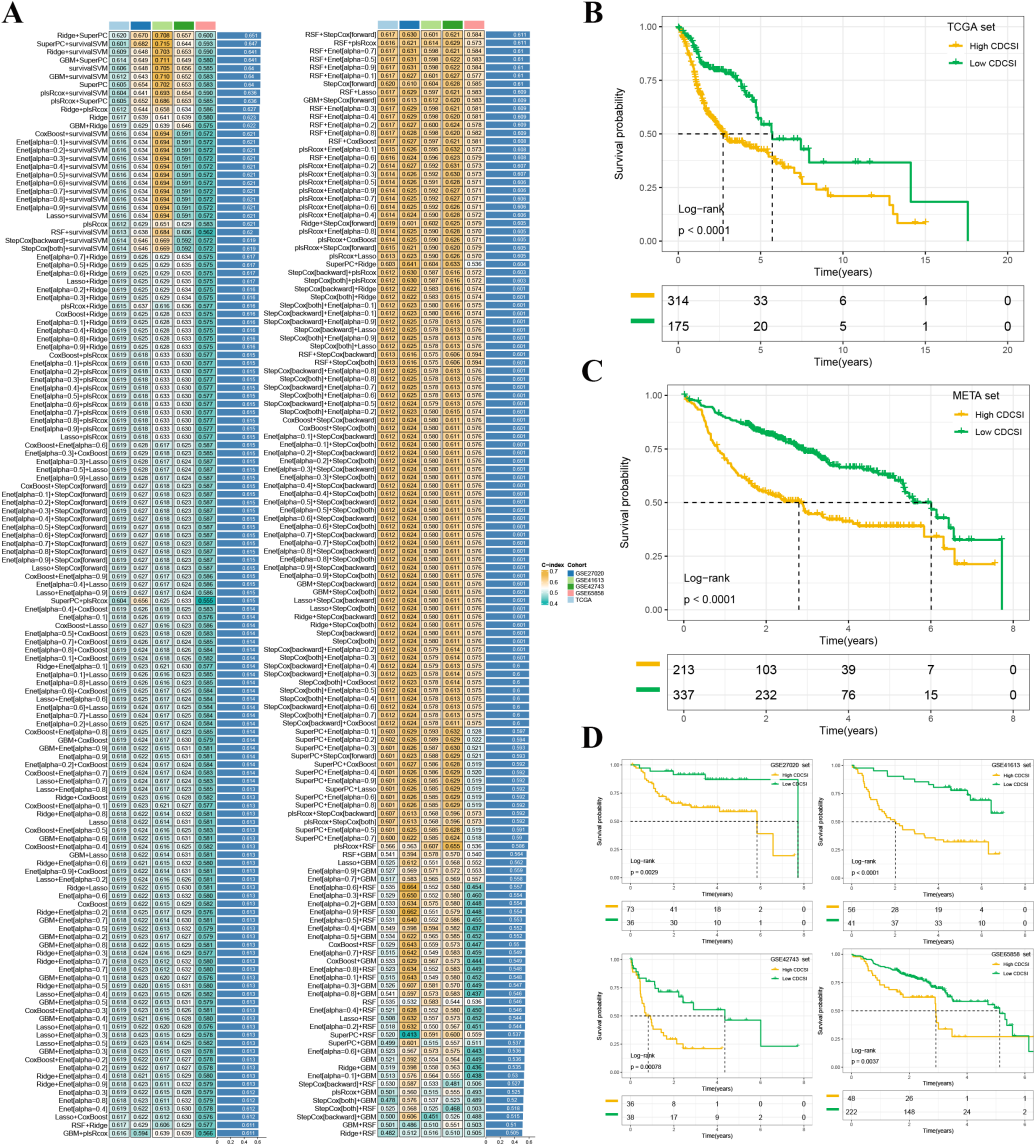
Construction of the cell death and cellular senescence index. **(A)** Heatmap showing the performance of 270 machine learning algorithm combinations for CDCSI construction. Model performance was evaluated using the average C-index across training and validation cohorts. The Ridge plus SuperPC algorithm achieved the highest average C-index of 0.651 and was selected as the optimal model. Six hub genes, including SERPINE1, THBS1, SRPX, FADD, SERINC3, and CSNK2A2, were included in the final CDCSI model. **(B–D)** Kaplan-Meier survival curves comparing overall survival between high- and low-CDCSI groups across multiple cohorts, including TCGA, META, GSE27020, GSE41613, GSE42743, and GSE65858. Patients with low CDCSI consistently exhibited better survival outcomes than those with high CDCSI.

Although CDCS subtype classification provided valuable group-level information, an individualized scoring system was needed for patient-level prognostic and therapeutic assessment. Therefore, we developed the CDCSI. Given that the CDCS2 subtype was associated with poor prognosis and resistance to multiple therapies, we first identified subtype-specific marker genes and intersected them with a curated set of 1,412 CDCS-related genes to generate candidate prognostic genes. Univariate Cox regression analysis was then performed across the TCGA and META cohorts to identify CDCS-related genes significantly associated with overall survival. Subsequently, predictive models were constructed using 270 combinations of machine learning algorithms, and their performance was evaluated by averaging the C-index across all datasets (**Figure 7A**). The combination of Ridge+SuperPC achieved the highest average C-index of 0.651. Using this model, six hub prognostic genes (SERPINE1, THBS1, SRPX, FADD, SERINC3, and CSNK2A2) were incorporated to generate the final CDCSI. CDCSI was calculated for each patient in all cohorts, revealing that higher CDCSI consistently correlated with worse clinical outcomes (**Figures 7B–D**).

### Validation of the cell death and cellular senescence index

**Figure 8:**
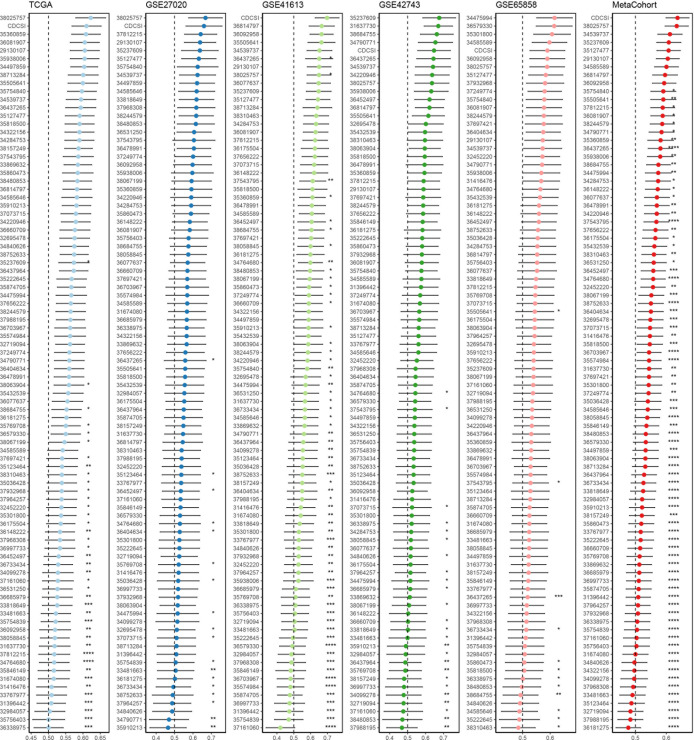
Comparison of CDCSI with published models. C-index comparison between CDCSI and 88 published HNSCC prognostic signatures across multiple independent cohorts. CDCSI demonstrated superior predictive performance, ranking first in the GSE41613 and META cohorts, second in the TCGA and GSE27020 cohorts, and fifth in the GSE42743 and GSE65858 cohorts. These results indicate that CDCSI has strong and stable prognostic performance compared with existing models.

**Figure 9:**
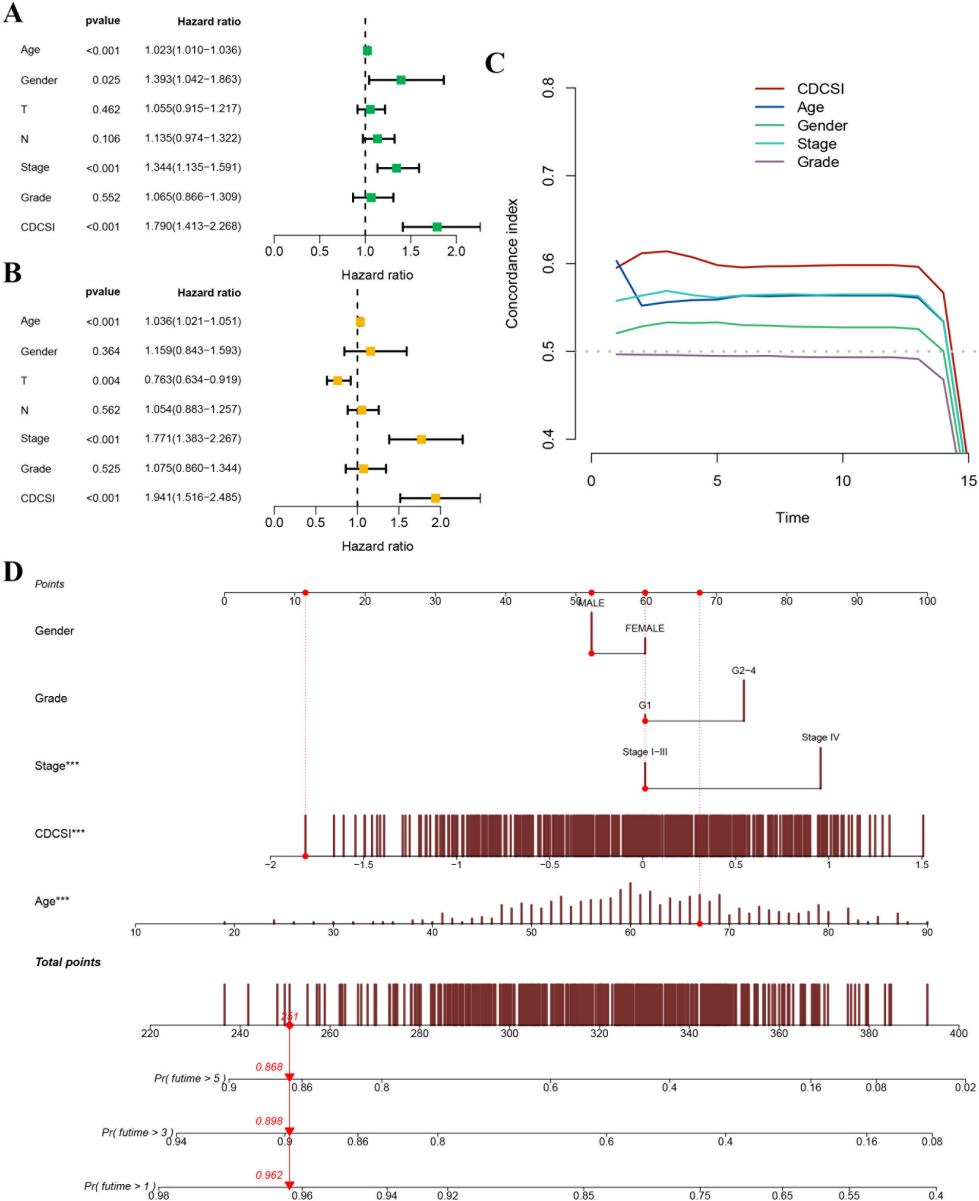
Comparison of CDCSI with clinical features. **(A)** Univariate Cox regression analysis evaluating the prognostic significance of CDCSI and conventional clinical variables. **(B)** Multivariate Cox regression analysis confirming that CDCSI is an independent prognostic factor. **(C)** C-index comparison between CDCSI and traditional clinical features, showing that CDCSI provides better predictive accuracy and stability. **(D)** Nomogram integrating CDCSI with key clinical variables to estimate individualized survival probability, thereby improving the potential clinical applicability of CDCSI.

To assess the prognostic robustness of CDCSI, we first calculated the C-index and benchmarked it against 88 previously published HNSCC prognostic signatures. CDCSI demonstrated superior performance, ranking highest in the GSE41613 and META cohorts, second in the TCGA and GSE27020 cohorts, and fifth in the GSE42743 and GSE65858 cohorts (**Figure 8**). To further evaluate its clinical relevance, univariate and multivariate Cox regression analyses were performed, confirming that CDCSI serves as an independent prognostic factor (**Figures 9A, B**). Comparison of C-index values also demonstrated that CDCSI outperformed traditional clinical features in terms of predictive accuracy and stability (**Figure 9C**). Finally, to facilitate clinical application and individualized risk assessment, we constructed a nomogram integrating CDCSI with key clinical characteristics. This tool allows for straightforward estimation of patient-specific survival probabilities and may support precision treatment decision-making (**Figure 9D**). Overall, these validation steps were crucial to establish CDCSI not only as a statistically robust prognostic signature but also as a clinically actionable index for HNSCC patient stratification.

### Correlation of CDCSI with therapeutic response

**Figure 10:**
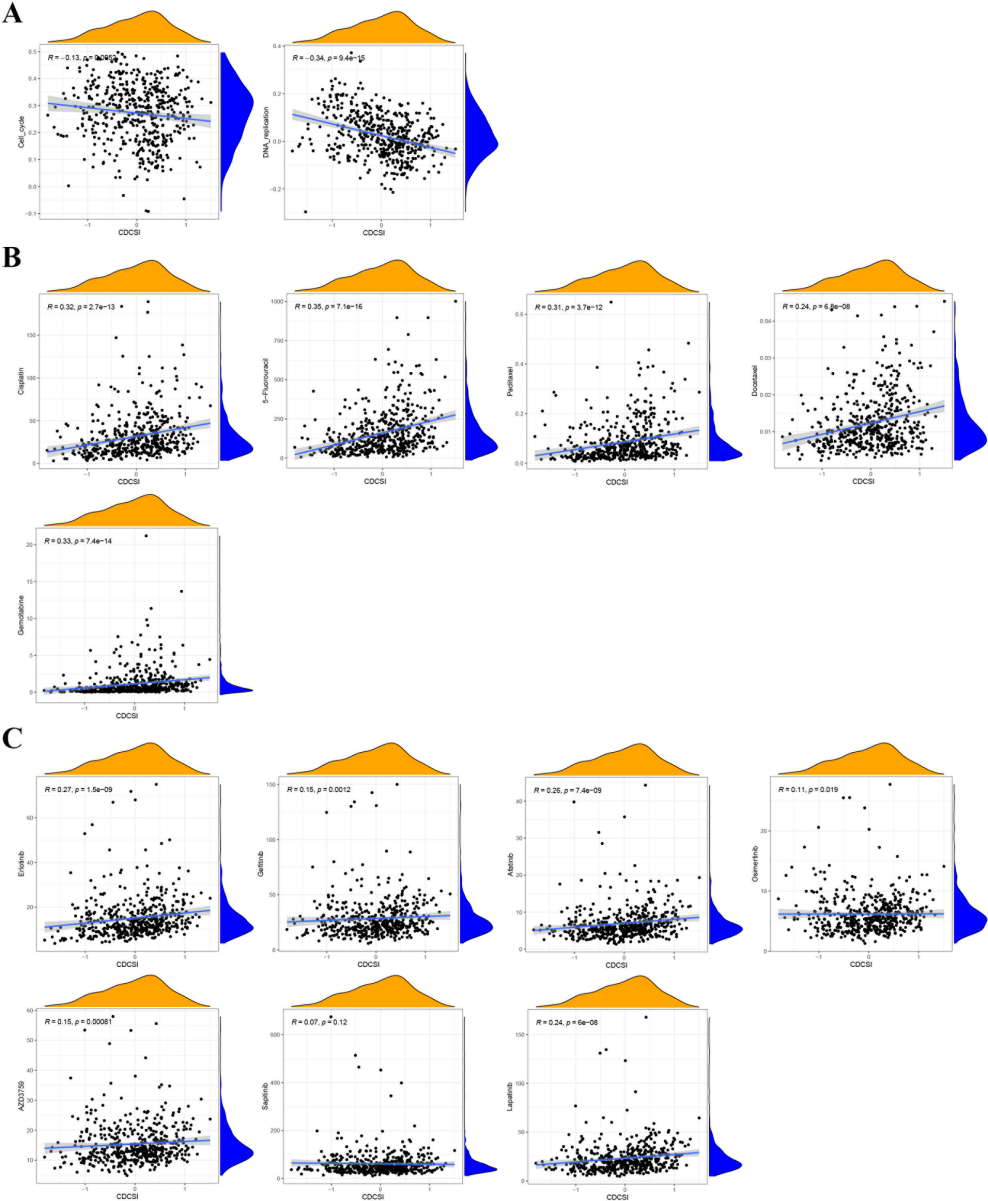
The relationship between CDCSI and response to chemotherapy, radiation, and EGFR-targeted treatment. **(A)** CDCSI exhibited a negative correlation with radiotherapy-related signatures, including cell cycle and DNA replication, suggesting that lower CDCSIs may indicate greater radiosensitivity. **(B)** Lower CDCSI were associated with reduced IC50 values for five standard chemotherapeutic agents (5-Fluorouracil, Cisplatin, Docetaxel, Gemcitabine, and Paclitaxel), indicating higher sensitivity to chemotherapy. **(C)** Lower CDCSI were associated with reduced IC50 values for seven EGFR-targeted drugs (Afatinib, AZD3759 (Zorifertinib), Erlotinib, Gefitinib, Lapatinib, Osimertinib, and Sapitinib), indicating higher sensitivity to targeted therapy.

**Figure 11:**
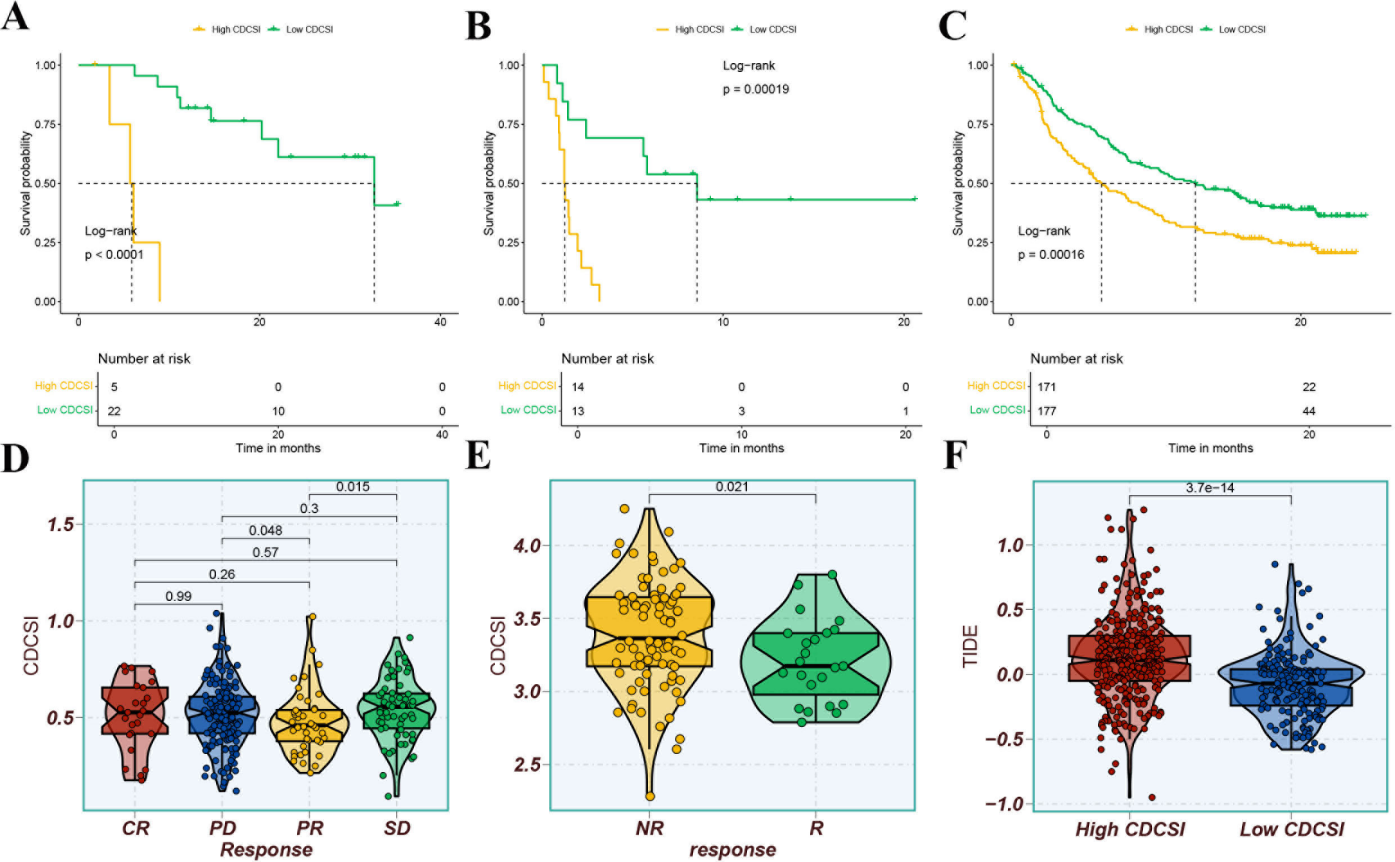
The relationship between CDCSI and response to immunotherapy. **(A–C)** Kaplan-Meier survival analyses in three independent immunotherapy cohorts, including GSE78220, GSE135222, and IMvigor210. Patients with low CDCSI showed better survival outcomes after immunotherapy. **(D)** Comparison of CDCSI among different clinical response groups in the IMvigor210 cohort. Patients achieving complete response or partial response had lower CDCSI than those with stable disease or progressive disease. **(E)** Comparison of CDCSI between responders and non-responders in the GSE91061 cohort. Responders exhibited lower CDCSI than non-responders. **(F)** TIDE analysis comparing immune evasion potential between high- and low-CDCSI groups. Low-CDCSI patients showed lower TIDE scores, indicating reduced immune dysfunction and exclusion and a higher likelihood of response to immune checkpoint blockade.

We next explored the relationship between CDCSI and treatment sensitivity. CDCSI exhibited a negative correlation with radiotherapy-related signatures, including cell cycle and DNA replication, suggesting that lower CDCSI may indicate greater radiosensitivity (**Figure 10A**). Similarly, lower CDCSI were associated with reduced IC50 values for five standard chemotherapeutic agents (5-Fluorouracil, Cisplatin, Docetaxel, Gemcitabine, and Paclitaxel) and seven EGFR-targeted drugs (Afatinib, AZD3759 (Zorifertinib), Erlotinib, Gefitinib, Lapatinib, Osimertinib, and Sapitinib), indicating higher sensitivity to both chemotherapy and targeted therapy (**Figures 10B, C**). Regarding immunotherapy, patients with low CDCSI demonstrated better survival across three independent immunotherapy cohorts (GSE78220, GSE135222, and IMvigor210), suggesting increased benefit from immune checkpoint blockade (**Figures 11A–C**). Furthermore, in the IMvigor210 cohort, CDCSI was lower in patients achieving complete or partial response (CR/PR) compared to those with progressive or stable disease (PD/SD), while in GSE91061, responders exhibited lower CDCSI than non-responders (**Figures 11D, E**). Consistently, the low CDCSI group corresponded with reduced TIDE scores, indicating lower immune suppression and immune evasion (**Figure 11F**). Taken together, these findings suggested that CDCSI is not only a prognostic index but also a potential predictor of therapeutic sensitivity. Low CDCSI may indicate greater benefit from radiotherapy, chemotherapy, EGFR-targeted therapy, and immunotherapy, whereas high CDCSI may identify patients with potential multi-treatment resistance.

### SHAP analysis for gene importance and individual contribution in CDCSI

**Figure 12:**
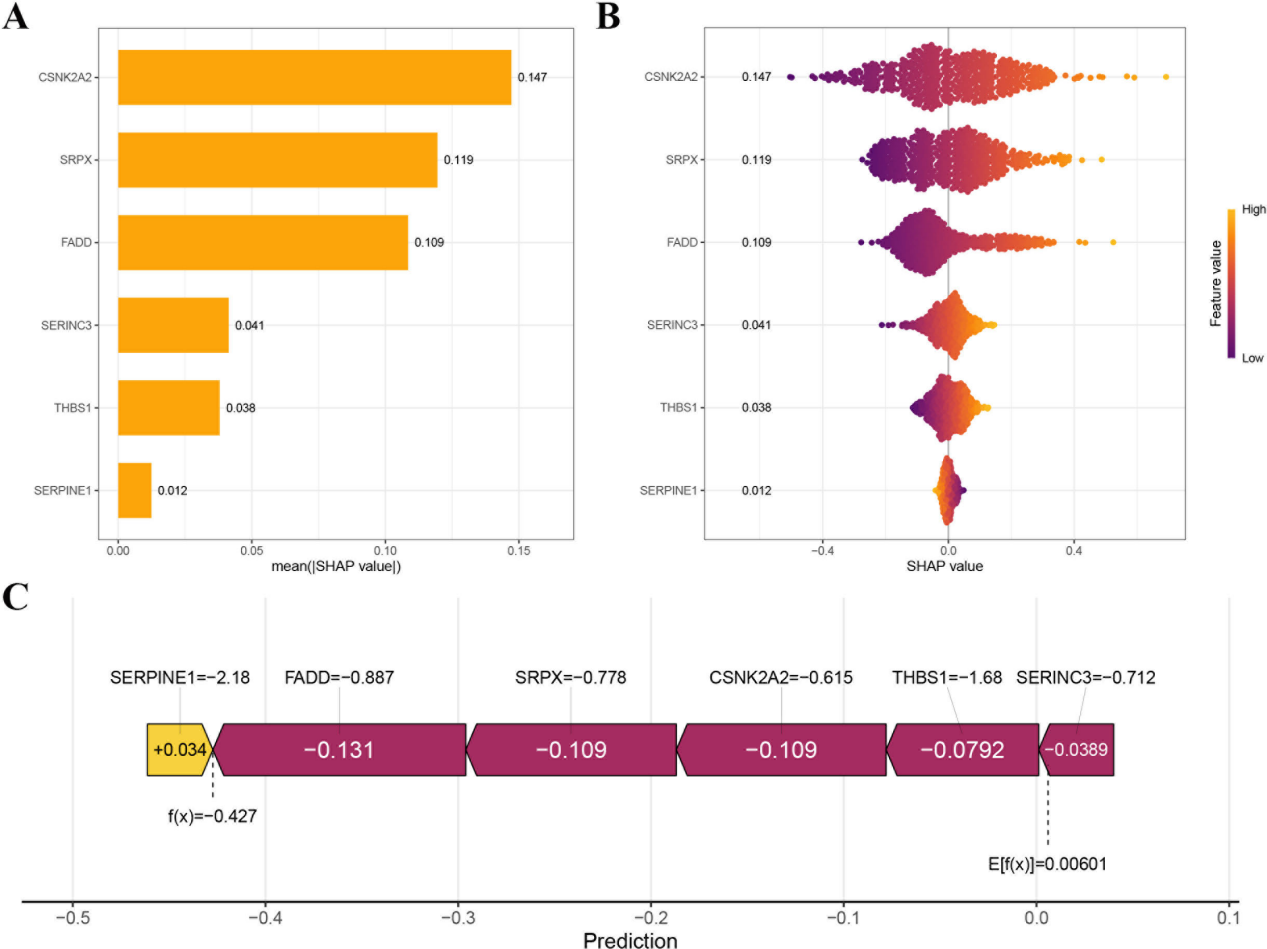
SHAP analysis for gene importance and individual contribution in CDCSI. **(A)** The gene importance ranking was derived from the average SHAP values, with higher values indicating greater influence on prognostic predictions. **(B)** The effect of individual gene expression on prognostic predictions. **(C)** A decomposition of the model prediction for individual patients, showing how the six genes collectively shift the baseline prediction toward the final outcome.

To enhance the interpretability of the CDCSI model, SHAP analysis was performed to quantify the contribution of each gene to model predictions. The gene importance ranking was derived from the average SHAP values, with higher values indicating greater influence on prognostic predictions (**Figure 12A**). Among the six genes, CSNK2A2 exhibited the highest contribution to the model output. **Figure 12B** illustrated the effect of individual gene expression on prognostic predictions. For example, higher expression of CSNK2A2 (yellow region) corresponded to increased SHAP values, indicating a higher likelihood of poor prognosis, whereas lower expression (purple region) was associated with decreased SHAP values and a more favorable prognosis. **Figure 12C** provided a decomposition of the model prediction for individual patients, showing how the six genes collectively shift the baseline prediction toward the final outcome, thereby offering molecular-level interpretability for CDCSI predictions.

### Single-cell and spatial transcriptomic validation

**Figure 13:**
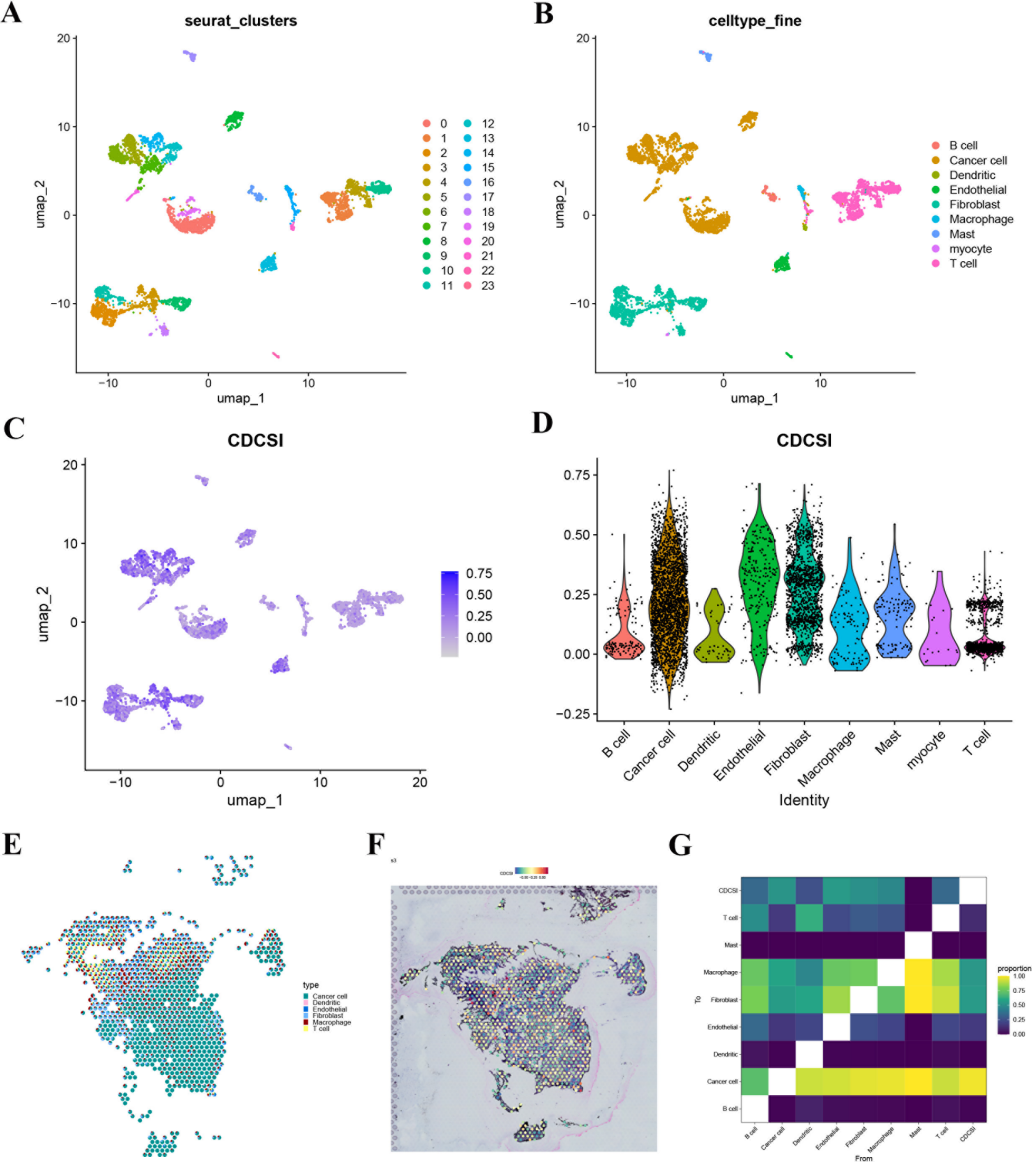
Single-cell and spatial transcriptomic validation. **(A)** UMAP visualization of single-cell RNA sequencing data showing 24 cell clusters. **(B)** Annotation of the 24 clusters into 9 major cell types. **(C)** UMAP visualization showing the distribution of CDCSI scores across single-cell populations. **(D)** Violin plot comparing CDCSI scores among different cell types. Tumor cells exhibited the highest CDCSI, indicating that CDCSI is mainly enriched in malignant cell populations. **(E)** Spatial transcriptomic visualization showing the distribution of CDCSI scores across spots. **(F)** Spatial localization of CDCSI-enriched regions, showing that high CDCSI was mainly distributed in tumor-enriched areas. **(G)** Colocalization analysis showing a positive correlation between tumor cell abundance and CDCSI scores, supporting the spatial association between CDCSI and malignant tumor regions.

To validate CDCSI at higher biological resolution, scRNA-seq and ST analyses were conducted. scRNA-seq data identified 24 clusters corresponding to 9 distinct cell types (**Figures 13A, B**). Using the ssGSEA algorithm, CDCSI was computed for each cell type. UMAP and violin plots revealed that tumor cells exhibited the highest CDCSI (**Figures 13C, D**). This result suggested that CDCSI mainly reflects malignant epithelial cell-related biological programs rather than being driven only by non-malignant stromal or immune components. Spatial transcriptomics analysis further confirmed these findings. CDCSI were calculated for each spatial spot, and UMAP visualization demonstrated higher scores localized within tumor regions (**Figures 13E, F**). Colocalization analysis indicated a strong positive correlation between tumor cells and CDCSI (**Figure 13G**), supporting the association between CDCSI and malignant cell populations within the tumor microenvironment.

### Validation of mRNA and protein expression levels *in vitro*

**Figure 14:**
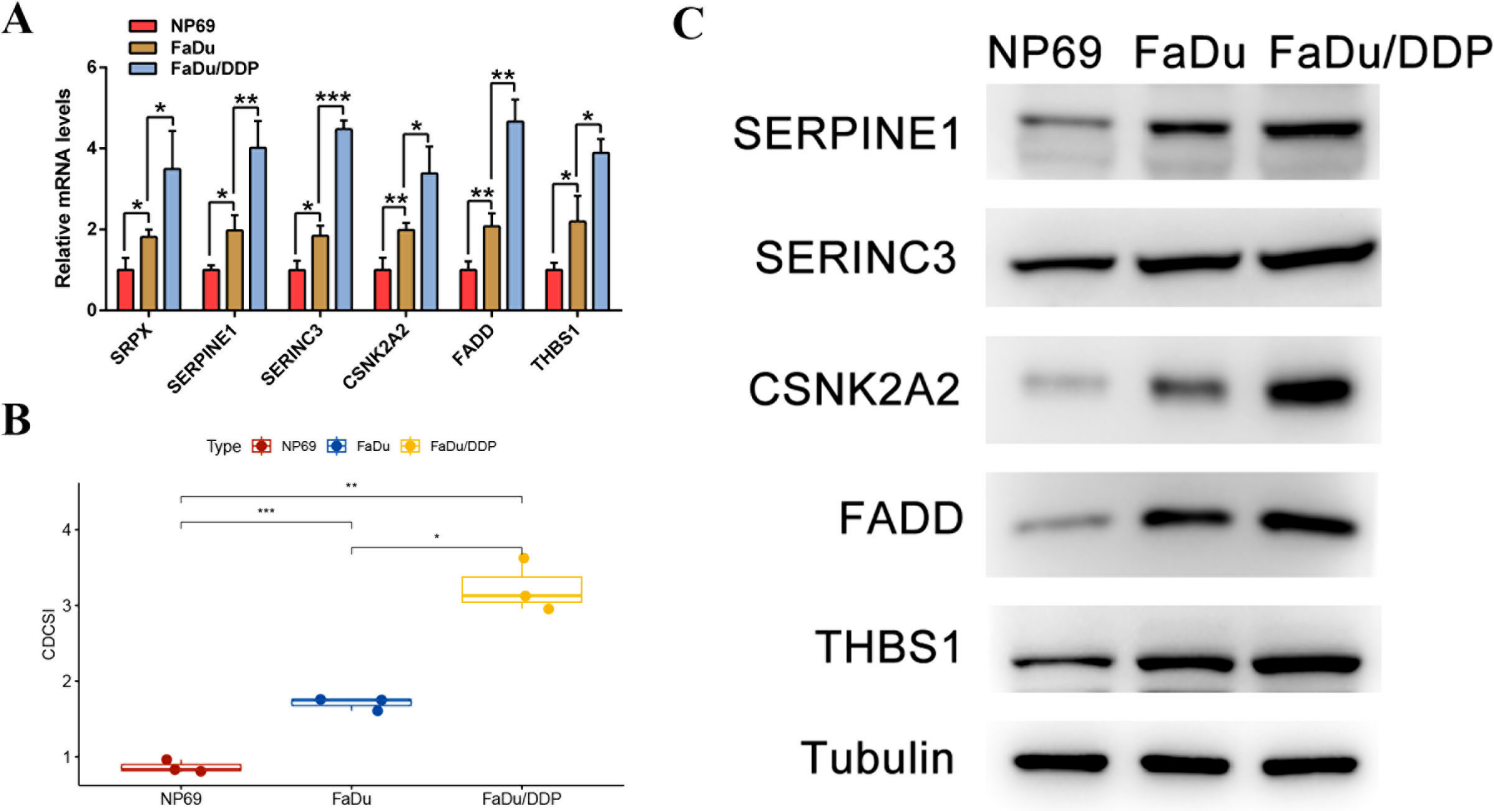
Validation of mRNA and protein expression levels *in vitro*. **(A)** The qPCR results demonstrated that mRNA expression levels of the six CDCSI genes (SERPINE1, SERINC3, CSNK2A2, FADD, THBS1, and SRPX) were highest in the cisplatin-resistant tumor cells, intermediate in the tumor cells, and lowest in normal cells. **(B)** The CDCSI calculated based on qPCR data was highest in cisplatin-resistant tumor cells, intermediate in tumor cells, and lowest in normal cells. **(C)** WB analysis confirmed protein-level expression patterns, showing elevated levels of five genes (SERPINE1, SERINC3, CSNK2A2, FADD, THBS1) in cisplatin-resistant tumor cells compared to tumor and normal cells.

Finally, qPCR and western blot analyses were performed to experimentally validate the expression patterns of CDCSI-related genes. Three cell lines were used: NP69 as a normal epithelial cell line, FaDu as a tumor cell line, and FaDu/DDP as a cisplatin-resistant tumor cell line. The qPCR results demonstrated that mRNA expression levels of the six genes (SERPINE1, SERINC3, CSNK2A2, FADD, THBS1, and SRPX) were highest in the cisplatin-resistant tumor cells, intermediate in the tumor cells, and lowest in normal cells (**Figure 14A**). Correspondingly, CDCSI calculated from the qPCR data mirrored this trend, validating the index at the transcriptional level (**Figure 14B**). WB analysis further confirmed protein-level expression patterns, showing elevated levels of five genes (SERPINE1, SERINC3, CSNK2A2, FADD, THBS1) in cisplatin-resistant tumor cells compared to tumor and normal cells (**Figure 14C**). These experimental results provided preliminary mRNA- and protein-level evidence supporting the biological relevance of CDCSI. The higher CDCSI observed in cisplatin-resistant cells also suggested a potential association between CDCSI and treatment resistance, although further functional experiments are required to clarify the causal roles of these genes in HNSCC progression and therapeutic resistance.

## Discussion

HNSCC was characterized by heterogeneity and therapeutic resistance, posing substantial challenges to effective clinical management (89). Although traditional staging systems had been widely used to guide treatment decision-making and surveillance strategies, they often failed to capture the variability in clinical outcomes among patients with the same disease stage (90). With advances in molecular biology and immunology, therapeutic options for HNSCC had become increasingly diverse (91). However, this expanding therapeutic landscape also highlighted the lack of reliable biomarkers for accurately predicting treatment responses and guiding personalized therapeutic strategies (92). RCD and CS were recognized as fundamental cancer hallmarks with profound effects on tumorigenesis, progression, and treatment sensitivity (4). Despite their biological significance, the integrated roles of CDCS-related characteristics in HNSCC remained unclear. Therefore, we systematically analyzed CDCS-related features, focusing on their expression profiles and interaction patterns in HNSCC. Based on these findings, we developed an individualized predictive model, termed CDCSI, to improve prognostic assessment, enhance the prediction of treatment responses, and facilitate more precise and personalized clinical decision-making.

In this study, we identified two distinct CDCS subtypes in HNSCC using multi-omics data and ten clustering algorithms. These subtypes exhibited different molecular characteristics, prognoses, and treatment responses, with CDCS2 showing broad resistance to radiotherapy, chemotherapy, targeted therapy, and immunotherapy. By analyzing marker genes associated with the CDCS2 subtype, we identified subtype-associated marker genes and integrated 270 machine learning algorithms to construct an individualized predictive model. The Ridge+SuperPC algorithm demonstrated the best performance, resulting in the development of CDCSI, which included six genes: SERPINE1, THBS1, SRPX, FADD, SERINC3, and CSNK2A2. We validated CDCSI across multiple methods, including clinical features, external datasets, and previously published models, confirming its superior predictive performance. Further analysis revealed that patients with low CDCSI were more likely to respond to radiotherapy, chemotherapy, EGFR-targeted therapy, and immunotherapy, whereas patients with high CDCSI exhibited stronger therapeutic resistance. The interpretability of CDCSI was enhanced through SHAP analysis, and its expression patterns were verified at single-cell and spatial transcriptomic levels, as well as through qPCR and WB for mRNA and protein expression. These findings underscored the value of CDCSI-based patient stratification in improving treatment selection accuracy and potentially enhancing clinical outcomes.

From a mechanistic perspective, most genes incorporated into the CDCSI appeared to converge on several biological processes highly relevant to HNSCC progression, including cell survival, invasive behavior, microenvironmental remodeling, and treatment resistance. CSNK2A2, FADD, THBS1, SERPINE1, and SRPX had each been linked to aggressive tumor phenotypes in prior studies, suggesting that the prognostic value of CDCSI might partly have arisen from its ability to capture a coordinated malignant program rather than the effect of a single gene alone. For example, CSNK2A2 was reported to promote proliferation, migration, angiogenesis, and tumor growth through activation of the NF-κB pathway, whereas FADD was implicated in squamous malignancies as a noncanonical oncogenic regulator associated with metastatic progression and poor survival (93–99). Likewise, THBS1 and SERPINE1 were both strongly connected to extracellular matrix remodeling, stromal activation, immune modulation, and invasive potential, indicating that they might have reflected an HNSCC phenotype characterized by enhanced tumor-stroma interaction and a more therapy-resistant microenvironment (100–107). SRPX, although mainly studied in glioma, was also associated with stemness, recurrence, and treatment resistance, further supporting the notion that this model might have captured conserved mechanisms of malignant plasticity and aggressiveness (108, 109). In this context, the enrichment of these genes in CDCSI suggested that a high-risk score might have represented a biological state marked by enhanced proliferative signaling, resistance to cell death, increased invasive capacity, and microenvironmental adaptation. In contrast, SERINC3 had not been well established as a classical cancer-driving gene and appeared to be more closely related to membrane lipid remodeling and antiviral innate immune responses (110, 111). However, its inclusion in the present model might still have been biologically meaningful, as genes that were not direct oncogenic drivers could nevertheless have reflected broader stress-response states, membrane dynamics, or immune-related processes relevant to tumor behavior. Taken together, these observations suggested that the CDCSI captured not only genes with recognized protumor functions but also genes that potentially represented the broader biological context of cell death, senescence, and tumor microenvironment interactions in HNSCC.

In the current treatment landscape of HNSCC, multimodal therapy remains the standard approach, with surgery followed by risk-adapted radiotherapy or chemoradiotherapy for locally advanced disease and platinum-based chemotherapy, cetuximab, and PD-1 blockade serving as major systemic options for recurrent or metastatic disease (112, 113). However, durable benefit remained limited in a substantial proportion of patients, and resistance to radiotherapy, chemotherapy, targeted therapy, and immunotherapy continued to be a major clinical challenge (114). Our finding that a high CDCSI was consistently associated with reduced sensitivity across these treatment modalities suggested that this index might have captured a shared biological state of treatment refractoriness rather than modality-specific resistance alone. Mechanistically, this interpretation was supported by the central role of the HNSCC tumor microenvironment in mediating broad therapeutic resistance. Previous studies had shown that immunosuppressive and stromal components of the HNSCC microenvironment, including TAMs, Tregs, MDSCs, and CAFs, could promote resistance to cisplatin, taxanes, cetuximab, radiotherapy, and immune checkpoint blockade through inflammatory signaling, extracellular matrix remodeling, metabolic reprogramming, and suppression of effective antitumor immunity (115–117). In parallel, contemporary immunotherapy studies had emphasized that only a subset of patients with HNSCC derived durable benefit from PD-1/PD-L1 inhibitors and that biomarker-driven strategies were urgently needed to improve patient selection and combination treatment design (115, 118). Thus, the association between high CDCSI and pan-therapeutic resistance might have reflected a more aggressive tumor state characterized by impaired cell-death responsiveness, senescence-associated microenvironmental remodeling, and immune exclusion, which together reduced vulnerability to both cytotoxic and immune-based treatments. From a clinical perspective, these findings suggested that CDCSI might have served not only as a prognostic indicator but also as a practical stratification tool for identifying patients less likely to benefit from conventional treatment paradigms and more likely to require biomarker-guided, combination, or microenvironment-targeted therapeutic strategies in HNSCC.

Compared with previous studies, our work had several notable strengths and distinctions. First, rather than focusing on a single RCD or CS alone, we investigated the integrated landscape of CDCS in HNSCC, thereby providing a more comprehensive framework for understanding tumor heterogeneity. Second, we incorporated multi-omics data from multiple molecular dimensions and applied a consensus strategy based on 10 clustering algorithms, which allowed us to capture CDCS-related heterogeneity more robustly while reducing potential bias introduced by any single clustering method. Third, the marker genes used for model construction were derived from the high-risk CDCS2 subtype and further filtered across multiple cohorts, which improved the stability and biological relevance of the selected features. Fourth, by integrating data from multiple independent cohorts and comprehensively evaluating 270 combinations of 10 machine learning algorithms, we established the CDCSI model with the best overall predictive performance, thereby enhancing its robustness, generalizability, and potential clinical utility.

Several limitations of this study should be acknowledged. First, this study was primarily based on retrospective data from public databases. Although CDCSI was trained and validated across multiple cohorts, the lack of an independent in-house cohort and the possibility that overfitting cannot be completely excluded remain limitations. Second, some public datasets lacked sufficient clinical and molecular annotations, which may have masked potential associations between CDCSI and certain variables. Third, although the expression of CDCSI-related genes was validated at both the mRNA and protein levels, additional functional and phenotypic experiments are still needed to clarify their biological roles. Therefore, further prospective studies and experimental validation are required to strengthen the clinical applicability of CDCSI.

## Conclusions

In conclusion, through the integration of comprehensive bioinformatics analyses and machine learning algorithms, we developed CDCSI as a robust and reliable signature for predicting prognosis, characterizing molecular features, and evaluating potential responses to radiotherapy, chemotherapy, targeted therapy, and immunotherapy in HNSCC. These findings suggested that CDCSI may provide an exploratory framework for individualized risk stratification and hypothesis generation regarding therapeutic response in HNSCC, but prospective clinical validation is required before clinical application.

## Data Availability

All results generated in this study can be obtained by contacting the corresponding authors on reasonable request. The complete code and critical data are available on github (https://github.com/JYfantast/HNSCC).

## Supplementary material

Supplementary Figure 1 **(A and B)** PCA demonstrated improved consistency and stability across datasets.

Supplementary Figure 2 **(A)** The CPI and gap statistical analysis. **(B)** Consensus heatmap for two clusters. **(C)** The silhouette score. **(D)** A clustering matrix based on consensus.

Supplementary Table 1 The 20 CDCS gene sets.

Supplementary Table 2 The primer sequences of the six genes (SERPINE1, SERINC3, CSNK2A2, FADD, THBS1, and SRPX).

Supplementary Table 3 The GO analysis for the CDCS2-related genes.

Supplementary Table 4 The KEGG analysis for the CDCS2-related genes.
