# Supplementary figures and images for: CDCSI: a machine learning-based interpretable cell death and cellular senescence index for prognosis improvement, immune landscape characterization, and therapeutic response prediction in head and neck squamous cell carcinoma
Source: Front Immunol. 2026 May 22;17:1848488. doi: 10.3389/fimmu.2026.1848488 (PMC13236947; doi:10.3389/fimmu.2026.1848488)

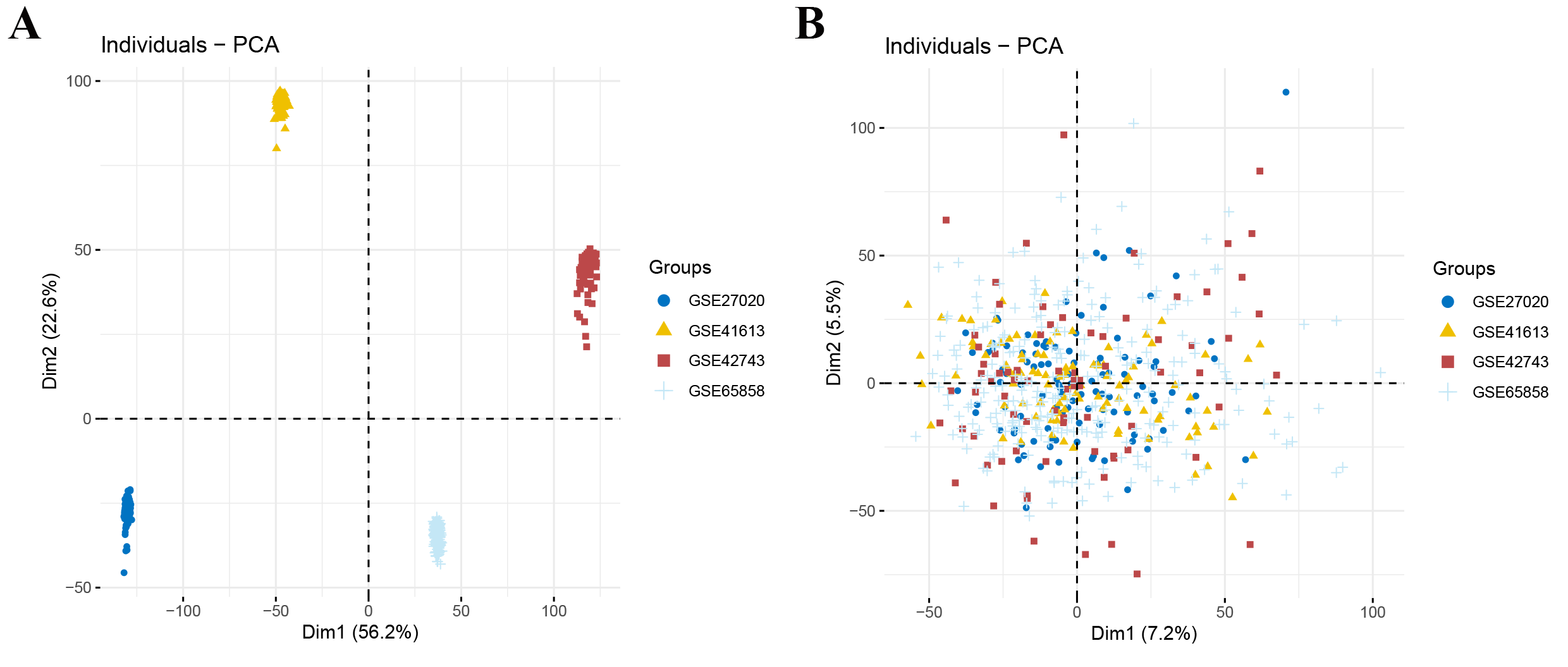

Supplement: Supplementary Figure 1 — (A and B) PCA demonstrated improved consistency and stability across datasets. [file Image1.tif]

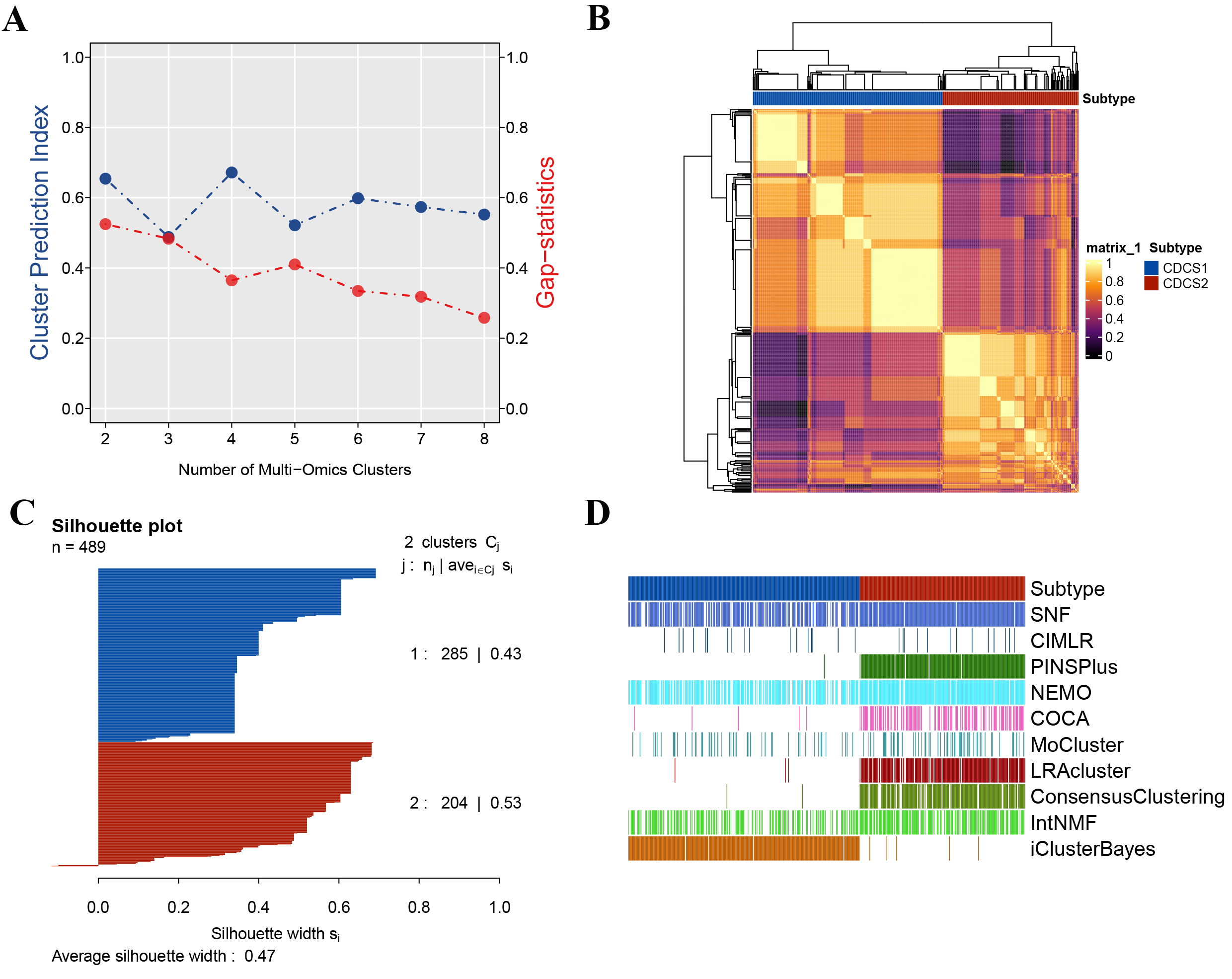

Supplement: Supplementary Figure 2 — (A) The CPI and gap statistical analysis. (B) Consensus heatmap for two clusters. (C) The silhouette score. (D) A clustering matrix based on consensus. [file Image2.tif]
